# VAPYRIN Marks an Endosomal Trafficking Compartment Involved in Arbuscular Mycorrhizal Symbiosis

**DOI:** 10.3389/fpls.2019.00666

**Published:** 2019-06-04

**Authors:** Laure Bapaume, Sabine Laukamm, Geoffrey Darbon, Corinne Monney, Felix Meyenhofer, Nadja Feddermann, Min Chen, Didier Reinhardt

**Affiliations:** Department of Biology, University of Fribourg, Fribourg, Switzerland

**Keywords:** arbuscular mycorrhiza, symbiosis, VAPYRIN, VAMP721, petunia hybrida, endosome

## Abstract

Arbuscular mycorrhiza (AM) is a symbiosis between plants and AM fungi that requires the intracellular accommodation of the fungal partner in the host. For reciprocal nutrient exchange, AM fungi form intracellular arbuscules that are surrounded by the peri-arbuscular membrane. This membrane, together with the fungal plasma membrane, and the space in between, constitute the symbiotic interface, over which nutrients are exchanged. Intracellular establishment of AM fungi requires the VAPYRIN protein which is induced in colonized cells, and which localizes to numerous small mobile structures of unknown identity (Vapyrin-bodies). In order to characterize the identity and function of the Vapyrin-bodies we pursued a dual strategy. First, we co-expressed fluorescently tagged VAPYRIN with a range of subcellular marker proteins, and secondly, we employed biochemical tools to identify interacting partner proteins of VAPYRIN. As an important tool for the quantitative analysis of confocal microscopic data sets from co-expression of fluorescent proteins, we developed a semi-automated image analysis pipeline that allows for precise spatio-temporal quantification of protein co-localization and of the dynamics of organelle association from movies. Taken together, these experiments revealed that Vapyrin-bodies have an endosomal identity with trans-Golgi features, and that VAPYRIN interacts with a symbiotic R-SNARE of the VAMP721 family, that localizes to the same compartment.

## Introduction

Arbuscular mycorrhiza (AM) represents a wide-spread symbiotic association of plants with a monophyletic group of fungal endosymbionts (AM fungi) that are collectively known as the Glomeromycotina in the order Mucuromycota (Spatafora et al., 2016). All AM fungi are obligate biotrophs, i.e., they rely on living host cells to complete their life cycle (Smith and Read, 2008). The establishment of AM requires fundamental reorganization of the host cells to allow intracellular accommodation of the fungal symbiont. The early stages of AM involve an infection structure known as the pre-penetration apparatus (PPA), which forms at the site of fungal attachment and hyphopodium formation (Genre et al., 2005, 2008). The PPA is thought to be a prerequisite for fungal infection, and it determines the site of penetration and the subsequent trajectory of hyphal growth through the cells. AM symbiosis culminates with the formation of the arbuscules, highly branched fungal structures that are separated from the surrounding host cytoplasm by the periarbuscular membrane (PAM). The fungal membrane, the PAM around it, and the periarbuscular space (PAS) in between (including the fungal cell wall), constitute the symbiotic interface, over which signals, and nutrients are exchanged (Harrison, 2012; Gutjahr and Parniske, 2013).

Reorganization of host cells during intracellular accommodation of AM fungi has been described in considerable detail. It involves changes in the organization of microtubules and actin (Genre and Bonfante, 1998, 1999), changes in plastid organization and dynamics (Fester et al., 2001), and a general multiplication of the cytoplasmic constituents, including mitochondria, ER and all other organelles. In addition, the nucleus becomes enlarged, conceivably as a result of the transcriptional induction of hundreds of AM-related genes, and due to endoreduplication (Genre et al., 2008; Carotenuto et al., 2019). In addition, mycorrhizal colonization involves ectopic cell divisions in the root cortex (Russo et al., 2019). These features indicate that host cells undergo fundamental reprogramming during symbiosis.

The genetic basis of the changes associated with the establishment of AM has been addressed with two complementary approaches: (i) Forward genetic screens to identify genes required for symbiosis, and (ii) transcript profiling to identify AM-induced genes followed by reverse genetic analysis of their function by gene knockouts or gene silencing. The first approach has been very successful, in particular to identify genes required for early symbiotic signaling (reviewed in Harrison, 2012; Gutjahr and Parniske, 2013; Oldroyd, 2013). These genes, most of which are constitutively expressed before and during symbiosis, constitute the common symbiosis signaling pathway (CSSP), which is also required for root nodule symbiosis (RNS). Reverse genetic analysis has been particularly successful in the identification of genes that function after mutual recognition, potentially in the establishment and functioning of AM symbiosis, but also at the earliest steps of pre-symbiotic communication (Kretzschmar et al., 2012). Among the hundreds of genes that are induced during AM (Güimil et al., 2005; Hohnjec et al., 2005; Fiorilli et al., 2009; Guether et al., 2009; Breuillin et al., 2010; Gallou et al., 2011; Tromas et al., 2012; Handa et al., 2015; Rich et al., 2017a; Sugimura and Saito, 2017; Vangelisti et al., 2018), only few have been functionally characterized. On the other hand, for many symbiotic functions in AM symbiosis, the genetic basis remains unknown.

An exceptional case among the essential genes in AM is the *VAPYRIN* gene. It has been discovered independently in two host species, *Medicago truncatula* and *Petunia hybrida*, in three research groups involving both, forward and reverse genetic strategies (Feddermann et al., 2010; Pumplin et al., 2010; Murray et al., 2011). *Vapyrin* mutants have been found to have an intact calcium spiking response, indicating that VAPYRIN acts downstream of the calcium signal (Murray et al., 2011). VAPYRIN is required for both AM and RNS, and can therefore be regarded as a common symbiosis gene. *VAPYRIN* expression is induced during AM, in contrast to the components of the CSSP, compatible with a role downstream of the CSSP (Feddermann et al., 2010; Pumplin et al., 2010). The VAPYRIN protein neither carries a signal peptide, nor does it have any predicted transmembrane domains, it would therefore be expected to reside in the cytoplasm. However, in both, *Medicago truncatula* and *Petunia hybrida*, VAPYRIN-GFP localizes to small mobile subcellular compartments (Feddermann et al., 2010; Pumplin et al., 2010), which we will further refer to as Vapyrin-bodies. The movement of Vapyrin-bodies is reminiscent of the movement of organelles such as Golgi stacks, which have been shown to exhibit stop-and-go movement as a result of their interactions with the ER-actin network (Nebenführ et al., 1999; Brandizzi et al., 2002; Pena and Heinlein, 2013). Interestingly, AM fungal infection involves the accumulation of multiple exocytic markers, including Golgi stacks and vesicles, at sites of hyphal progression, indicating that intracellular accommodation of AM fungi requires active membrane dynamics (Genre et al., 2012).

VAPYRIN consists of two domains that are known as protein:protein interaction domains, an N-terminal VAMP-associated protein (VAP) domain [also known as major sperm protein (MSP) domain], and a C-terminal ankyrin (ANK) domain with 11 ankyrin repeats (Feddermann et al., 2010; Feddermann and Reinhardt, 2011). Both domains are known to interact with membrane proteins. In the case of the VAP domain, the name stands for vesicle-associated membrane protein (VAMP)-associated protein, hence, proteins with a VAP domain may associate with vesicles (Lev et al., 2008). On the other hand, the ANK domain is known to bind to integral membrane proteins such as ion channels and other membrane-resident proteins (Michaely et al., 2002; Mosavi et al., 2004).

The similarity of the expression patterns of VAPYRIN and the exocyst complex component EXO70I, and the fact that VAPYRIN can interact physically with EXO70I, indicated that Vapyrin-bodies may be involved in secretion (Zhang et al., 2015). Secretion involves vesicles carrying internal cargo or membrane constituents that become integrated in specific target membranes (Surpin and Raikhel, 2004). In cells with arbuscules, the default target membrane for secretion is the PAM that surrounds the fungal arbuscule and controls nutrient fluxes between both partners (Pumplin et al., 2012). However, apart from this information, the identity and function of the Vapyrin-bodies has remained largely elusive.

Here, we explore the identity and cellular function of the Vapyrin-bodies with biochemical methods and with co-localization experiments. We used a wide array of fluorescently labeled subcellular marker proteins as reference for co-localization studies, and we used a semi-automated bioinformatics pipeline to quantify co-localization and association of Vapyrin-bodies with a variety of subcellular compartments. This analysis revealed that Vapyrin-bodies have endosomal characteristics, and that they are associated with Golgi stacks and move together throughout the cytoplasm. The velocity of the Vapyrin-bodies and their association with the ER suggest that they are actively transported. A split-ubiquitin interaction screen in yeast identified a VAPYRIN-interacting protein, VAMP721, which is related to symbiotic VAMPs in *M. truncatula*. Taken together, our results indicate that Vapyrin-bodies have a mixed identity with trans-Golgi/endosomal characteristics which are compatible with a role in transport and secretion.

## Results

### Characterization of VAPYRIN-Bodies in *P. hybrida, N. benthamiana*, and *A. thaliana*

In order to systematically explore the identity of the VAPYRIN-bodies, we sought for an amenable expression system in which VAPYRIN localization could be studied in combination with a diverse panel of subcellular marker proteins. Infiltration of tobacco leaves (*Nicotiana benthamiana*) with *Agrobacterium tumefaciens* (agro-infiltration) is an established procedure for transient gene expression and subcellular localization studies (Leuzinger et al., 2013). The advantage of this method is the relatively rapid procedure (few days until readout), and its versatility, since different combinations of proteins can easily be co-expressed via a single infiltration.

**Figure 1:**
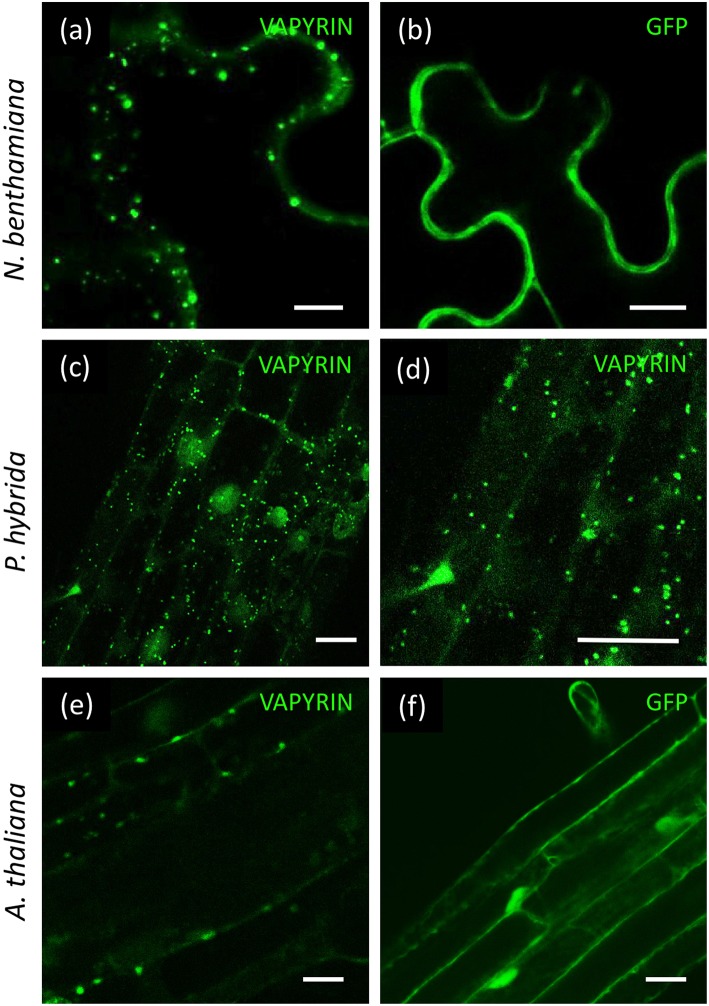
Localization of VAPYRIN-GFP in *N. benthamiana, P. hybrida*, and *A. thaliana*. **(a)** *N. benthamiana* leaves transformed with VARYIN-GFP by agro-infiltration exhibit mobile Vapyrin-bodies. **(b)** Free GFP in *N. benthamiana* leaves reveal cytoplasmic localization. **(c,d)** Localization of VAPYRIN-GFP to mobile Vapyrin-bodies in stably transformed *P. hybrida* roots. **(e)** Roots of stably transformed *A. thaliana* exhibit immobile Vapyrin-bodies. **(f)** Free GFP in stably transformed *A. thaliana* roots exhibits cytoplasmic localization. Size bar: 10 μm.

In order to test whether Vapyrin-bodies are formed in agro-infiltrated leaves, we transformed tobacco with VAPYRIN-GFP, and with free GFP as a reference (Figure 1). VAPYRIN-GFP was localized to mobile subcellular compartments (Figure 1a; Movie S1), unlike free GFP which exhibited a general cytoplasmic fluorescence (Figure 1b). The mobile dots had a similar appearance as in transgenic hairy roots of petunia (*P. hybrida*) (Feddermann et al., 2010) and *M. truncatula* (Pumplin et al., 2010), or as in stably transformed petunia roots expressing a VAPYRIN-GFP fusion (Figures 1c,d). On this basis, we consider tobacco leaves as a reliable model to study localization and movement of Vapyrin-bodies.

*Arabidopsis thaliana* is the preferred plant model system for cell biological studies due to the availability of many tools and resources, including the so-called Wave lines, which express a diverse range of fluorescently labeled marker proteins for various subcellular compartments (Geldner et al., 2009). To test the suitability of *Arabidopsis* for subcellular localization studies with VAPYRIN, we introduced VAPYRIN-GFP into *A. thaliana* by stable transformation. As in petunia, *Medicago* and tobacco, VAPYRIN-GFP in *Arabidopsis* roots was localized to small subcellular compartments (Figure 1e), unlike free GFP that exhibited a general cytosolic localization (Figure 1f). However, in contrast to the other model systems, the Vapyrin-bodies in *Arabidopsis* did not significantly move within the cells (Movie S2). Hence, some essential components may be missing in *Arabidopsis*, consistent with the fact that this non-symbiotic species has lost many AM-related genes (Delaux et al., 2014; Favre et al., 2014; Bravo et al., 2016). Since *Arabidopsis* did not show normal mobile Vapyrin-bodies, it was excluded from further experiments, and instead agro-infiltrated tobacco leaves were used for further characterization of Vapyrin-bodies.

### Both Domains of VAPYRIN Localize to VAPYRIN-Bodies

**Figure 2:**
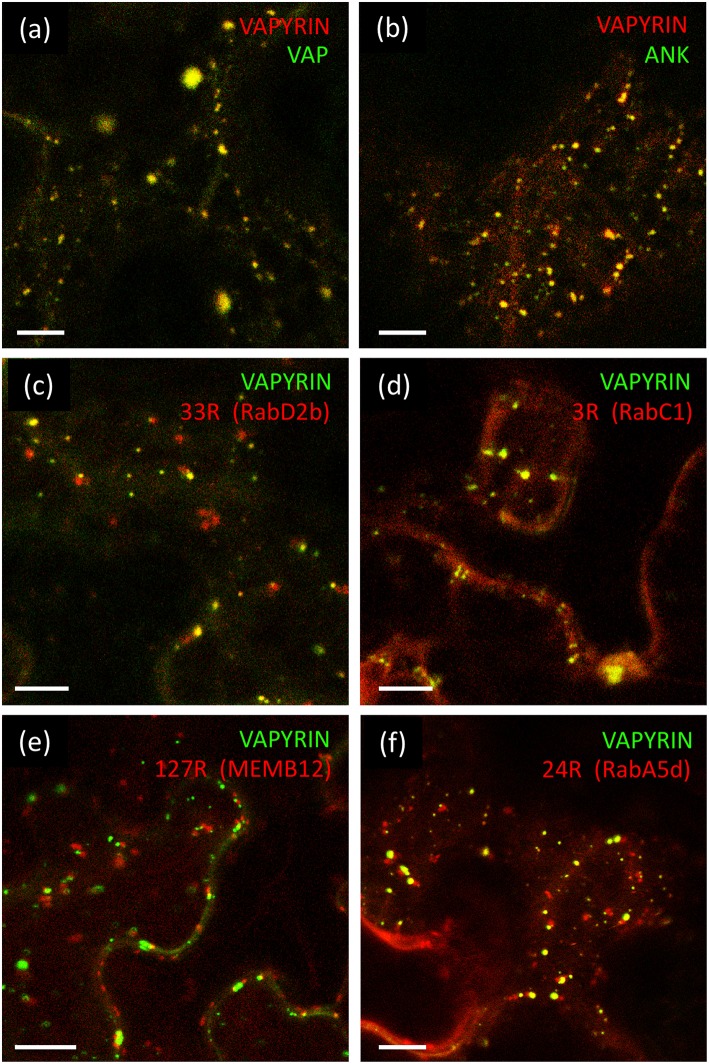
Localization of VAPYRIN protein domains and co-localization with Wave marker proteins. **(a)** Fluorescently tagged VAP domain (VAP-GFP) co-localizes with full-length VAPYRIN (VAPYRIN-RFP) on Vapyrin-bodies. **(b)** Fluorescently tagged ankyrin domain (ANK-GFP) co-localizes with full-length VAPYRIN (VAPYRIN-RFP) on Vapyrin-bodies. **(c)** Co-localization of the endosomal marker mCherry-RabD2b with VAPYRIN-GFP. **(d)** Co-localization of the post-Golgi/endosomal marker mCherry-RabC1 with VAPYRIN-GFP. **(e)** Association but no co-localization of the Golgi marker mCherry-MEMB12 with VAPYRIN-GFP. **(f)** Co-localization and association of the endosomal/recycling endosome marker mCherry-RabA5d with VAPYRIN-GFP. Size bar: 10 μm. See Figures S1, S2, S4 for pictures of the separate channels.

The VAP domain and the ANK domain are both known to interact with integral membrane proteins (Lev et al., 2008; Bennett and Healy, 2009; Cunha and Mohler, 2009), hence the VAPYRIN protein, which does not carry any recognizable features for a membrane localization, is likely to bind to the cytoplasmic surface of the membranes that surround the Vapyrin-bodies. In order to explore which of the two domains of VAPYRIN is responsible for the association with Vapyrin-bodies, they were both fused separately to GFP for localization experiments (VAP-GFP and ANK-GFP) and co-expressed with full-length VAPYRIN (VAPYRIN-RFP). In both cases, the truncated proteins were localized to small mobile compartments, and the co-localization with VAPYRIN-RFP identified them as Vapyrin-bodies (Figures 2a,b; Figures S1a,b). The overlap was equivalent to co-expression of two full length versions of VAPYRIN tagged with GFP and RFP, respectively (Figure S1c). Hence, both separate domains are sufficient on their own to mediate correct localization to Vapyrin-bodies.

### Vapyrin-Bodies Have Endosomal Characteristics

Small subcellular compartments like the Vapyrin-bodies may represent endosomal compartments in the endo- or exocytotic route of subcellular trafficking (Surpin and Raikhel, 2004). In order to explore the identity of VAPYRIN-bodies, a panel of Wave markers was employed for different endosomal subtypes, and intermediates in cellular trafficking and secretion (Geldner et al., 2009). Many of these markers involve highly conserved regulatory ARF and RAB GTPases that mark various trafficking compartments (Nielsen et al., 2008) (Table S1).

First, we employed the endosomal marker mCherry-RabD2b (**Wave 33R**) together with VAPYRIN-GFP (Figure 2c; Figure S2a; Movie S3). These two fluorescent protein fusions co-localized to a significant degree (Figure 2c), suggesting that Vapyrin-bodies have endosomal characteristics. An endosomal marker with a more narrow specificity for post-Golgi endosomal elements, RabC1 (**Wave 3R**) (Geldner et al., 2009), also exhibited a high degree of co-localization with VAPYRIN-GFP, indicating that VAPYRIN-bodies have post-Golgi identity (Figure 2D; Figure S2b; Movie S4).

### Association of Vapyrin-Bodies With Golgi and Recycling Endosome Markers

In order to further address the relationship between Vapyrin-bodies and the Golgi apparatus, we tested the marker MEMB12 (**Wave 127R**), which encodes a SNARE protein that specifically localizes to Golgi stacks (Uemura et al., 2004). Co-expression of VAPYRIN-GFP with mCherry-MEMB12 produced a conspicuous localization pattern. The rate of co-localization events was very low, but in many cases, close associations between Vapyrin-bodies and MEMB12-labeled compartments were observed (Figure 2e; Figures S2c, S3). Such associations remained stable over extended periods of time and were characterized by a constant short distance between the two compartments (Movie S5). These results indicate that a subpopulation of the VAPYRIN-bodies is closely associated with the Golgi apparatus. Hence, these observations are compatible with an identity of Vapyrin-bodies as elements of the trans-Golgi network (TGN). An identity as TGN was also supported by the partial colocalization of Vapyrin with the TGN marker SYP61 (Drakakaki et al., 2012; Hachez et al., 2014) (Figure S4a).

TGN elements converge with recycling endosomes (RE) in anterograde trafficking from the Golgi (Surpin and Raikhel, 2004). Hence, we tested whether VAPYRIN-GFP co-localized with the RE marker RabA5d (**Wave 24R**). Indeed, significant co-localization was observed (Figure 2f; Figure S4b; Movie S6), indicating that the identity of Vapyrin-bodies extended from the TGN-domain into the RE domain. A second marker for RE compartments, RabA1g (Wave 129R), only showed minimal co-localization with VAPYRIN-GFP (Figure S4c), indicating that RE represent a heterogenous category of compartments.

Golgi stacks are known to be closely associated with the ER and to move along ER strands in a stop-and-go mode (Pena and Heinlein, 2013). Given the TGN identity of Vapyrin-bodies, we tested the relationship of VAPYRIN-bodies with the ER by co-expression of VAPYRIN-RFP with GFP carrying an ER-specific localization signal (GFP-HDEL). In general, Vapyrin-bodies were closely associated with ER strands and in addition, they moved along ER strands (Figure S5). Based on these results, we suggest that Vapyrin-bodies feature TGN and RE identity.

### Vapyrin-Bodies Have no Pre-vacuolar, Vacuolar, or Autophagosomal Identity

**Figure 3:**
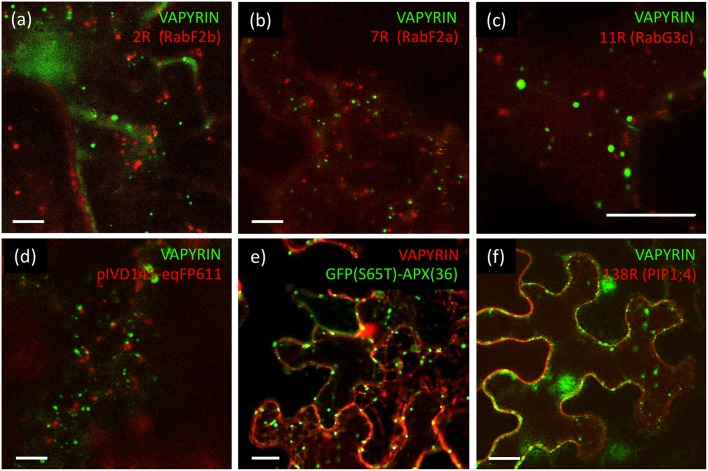
Lack of co-localization of VAPYRIN with markers for the vacuolar trafficking pathway and other subcellular compartments. **(a)** Co-expression of the late endosomal/prevacuolar marker mCherry-RabF2b with VAPYRIN-GFP reveals no co-localization. **(b)** Co-expression of the late endosomal/prevacuolar marker mCherry-RabF2a with VAPYRIN-GFP reveals no co-localization. **(c)** Co-expression of the late endosomal/prevacuolar marker mCherry-RabG3c with VAPYRIN-GFP reveals no co-localization. **(d)** Co-expression of the mitochondrial marker pIVD14s-eqFP611 with VAPYRIN-GFP reveals no co-localization. **(e)** Co-expression of the peroxisomal marker GFP(S65T)-APX(36) with VAPYRIN-RFP reveals no co-localization. **(f)** Co-expression of the plasma membrane marker PIP1;4-RFP with VAPYRIN-GFP reveals no co-localization. Size bar: 10 μm. See Figures S6, S7 for pictures of the separate channels.

In plant cells, anterograde trafficking comprises two main directions, the route toward the vacuole, and secretion to the apoplast across the plasma membrane (Surpin and Raikhel, 2004). In the case of mycorrhizal cells, the latter route becomes diverted to the symbiotic interface around the arbuscules (Pumplin et al., 2012). In order to distinguish between these two routes, we employed several markers that highlight the vacuolar pathway. RabF2b (**Wave 2R**) and RabF2a (**Wave 7R**) highlight multivesicular bodies (MVB), late endosomes, and prevacuolar compartments (Geldner et al., 2009). These markers did not show significant co-localization with VAPYRIN-GFP (Figures 3a,b; Figures S6a,b), neither did a third late endosomal/vacuolar marker, RabG3c (**Wave 11R**) (Figure 3c; Figure S6c), that has been detected in the vacuolar proteome (Carter et al., 2004). In addition, we tested two vacuolar aquaporins, gamma-TIP, and delta-TIP, that localize to a subdomain of the tonoplast (Saito et al., 2002), and which did not show any co-localization with Vapyrin (data not shown).

Finally, no co-localization was observed of VAPYRIN-RFP with the autophagic marker GFP-ATG8a (data not shown), which localizes to autophagosomes (Zhuang et al., 2017), small prevacuolar compartments implicated in degradation of cellular constituents (Michaeli et al., 2016). We also employed markers for mitochondria and peroxisomes to test whether they interacted with Vapyrin-bodies. The mitochondrial marker pIVD145-eqFP611 (Forner and Binder, 2007) did not at all co-localize or associate with VAPYRIN-GFP (Figure 3d; Figure S7a), nor did the peroxisomal marker GFP(S65T)-APX(36) (Forner and Binder, 2007) (Figure 3e; Figure S7b). Furthermore, no co-localization was detected with the plasma membrane marker PIP1;4 (**Wave 138**) (Figure 3f; Figure S7c). Taken together, these results show that Vapyrin-bodies are not intermediates in the trafficking route toward the vacuole, nor are they part of an autophagic pathway.

### Quantitative Analysis of Protein Co-localization and Compartment Association

Characterization of subcellular compartments by protein co-localization studies requires detailed spatio-temporal image analysis. Selection of representative images and counting of co-localizing structures by visual inspection may not always be sufficient for a quantitative analysis of such phenomena. In addition, individual images cannot reveal dynamic aspects that can only be observed in movies. Hence, we developed a semi-automated bioimage-informatics pipeline based on the Kalaimoscope MotionTracker software (Kalaidzidis et al., 1997; Rink et al., 2005; Collinet et al., 2010) (http://www.kalaimoscope.com/science.html). This tool uses specialized algorithms to process movies, involving global object recognition, establishment of movement tracks over time, and quantification of various parameters related to object area and movement tracks. Quantification from movies by movement tracks allows more consistent analysis than assessment of individual images. We used this tool to quantify co-localization and object association with a range of Wave markers and additional subcellular marker proteins.

**Figure 4:**
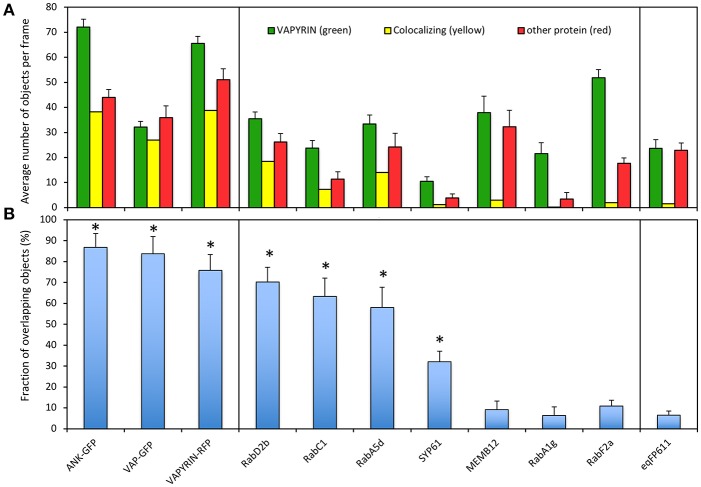
Quantification of VAPYRIN co-localization with various subcellular markers. **(A)** Fluorescent objects were detected by MotionTracker and assessed for co-localization by scoring the frequency of a 50% area overlap between adjacent objects in the two channels (see Figure S8 and Materials and Methods). The average number of objects per frame in the VAPYRIN channel (green), the other channel (red), and the number of colocalizing objects (yellow) are indicated. **(B)** The degree of co-localization was calculated by the number of co-localizing objects relative to the number of objects in the channel with fewer objects. Error bars represent standard deviations from 15 to 35 frames with an average of 31 objects per frame. Asterisks indicate pairs of markers that showed significant co-localization according to Tukey HSD *post-hoc* test (Table S2).

To quantify the degree of co-localization, we first determined the number of objects in the red and the green channel, and the number of colocalizing objects defined by an overlap of at least 50% of their area at half maximum intensity (Figure S8; Figure 4A). In cases, in which the number of objects in the two channels is different (for example due to a lower expression level of one of the two fluorescent marker proteins), the degree of overlap is limited by the channel with fewer objects. We defined the degree of colocalization as the number of overlapping objects relative to the number in the channel with fewer objects, because colocalization can only be defined for objects that have a signal in both channels. This has the disadvantage that less objects with the more abundant marker can be assessed.

To calibrate the quantification pipeline with positive controls, we used co-localization of VAPYRIN-RFP with ANK-GFP and VAP-GFP, which exhibited an indistinguishable subcellular localization pattern (Figure 1). In addition, VAPYRIN-GFP was co-expressed with VAPYRIN-RFP as a positive control. These positive controls yielded co-localization coefficients in the range of 75–90% (Figure 4B). As a negative control, the mitochondrial marker eqFP611 was used, which exhibited a co-localization coefficient of <5% (Figure 4B). These results show that this methodology allows to assign quantitative co-localization coefficients in the range of 5–90%.

Using this semi-automated quantification pipeline, we determined the level of co-localization of VAPYRIN-GFP with the following subcellular markers: RabD2b, RabC1, RabA5d, SYP61, MEMB12, RabA1g, and RabF2a. Consistent with the visual interpretation from individual representative images (Figures 2, 3; Figures S1–S4), the analysis revealed significant co-localization of VAPYRIN with RabD2b, RabC1, and RabA5d, whereas no significant co-localization resulted from MEMB12, RabA1g, and RabF2a (Figure 4B; Table S2). SYP61 took an intermediate position (Figure 4B; Table S2). Statistical analysis by Tukey HSD *post-hoc* test clearly distinguished significantly co-localizing markers (marked with asterisks in Figure 4B) from the rest (Table S2). This co-localization analysis confirms the conclusion that Vapyrin-bodies have a dual identity as TGN and RE.

**Figure 5:**
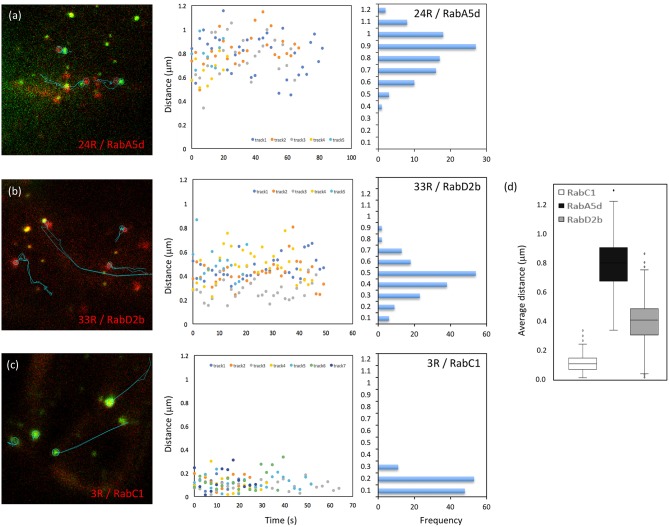
Distance between Vapyrin-bodies and associated endosomal compartments. Paired objects were identified in cells co-expressing Vapyrin-GFP and mCherry-RabA5d **(a)**, mCherry-RabD2b **(b)**, or mCherry-RabC1 **(c)**. Object pairs identified in a minimum of 10 consecutive frames were used for the measurement of the distance between their centers over time. Distances were plotted over time (middle), and the corresponding frequency distributions were determined from the same data (right). **(d)** Average distances of approximately 0.4 μm (RabD2b) and 0.8 μm (RabA5d) indicate non-random associations. The small distance of 0.1–0.2 μm for RabC1 indicates *bona fide* co-localization (compare with Figures 2, 4). All three distances in **(d)** were significantly different (*t*-test; *p* < 0.001).

Besides MEMB12, which exhibited association rather than co-localization with Vapyrin-bodies (Figures S2c, S3), two additional markers (RabD2b and RabA5d) showed association with Vapyrin-bodies in addition to co-localization (Figures 2c,f). This phenomenon was quantified using the movies used for co-localization analysis (Figure 5). First, paired tracks of objects in the red and green channel were identified and their tracks analyzed over multiple time frames (Figures 5a–c, left). Then the distance between the associated objects in the two channels was determined over time (Figures 5a–c, middle). Secondly, the distance distribution was derived from the same data set (Figures 5a–c, right), and the average distance between the objects was calculated (Figure 5d). This analysis showed that the distance between Vapyrin-bodies and the other endosomal compartments was within characteristic windows for each pair of markers. For example, for VAPYRIN-GFP and mCherry-RabA5d, the average distance was 0.79 ± 0.17 μm (median = 0.80 μm), whereas for VAPYRIN-GFP and mCherry-RabD2b, the distance was 0.40 ± 0.16 μm (median = 0.41 μm) (Figure 5d). This analysis shows that the two compartments were not associated in a random fashion. For comparison, the co-localizing marker RabC1 (**Wave 3R**) yielded an average distance between the two channels of 0.12 ± 0.07 μm (median = 0.11 μm) (Figure 5d). These small values are below the threshold of optical resolution for confocal microscopy, and are consistent with the conclusion that VAPYRIN and RabC1 co-localize on the same objects (compare with Figure 2d). Student's pairwise *t*-test revealed that the differences in the average distances of the three pairs (Figure 5d) were highly significant (*p* < 0.001). Taken together, these results suggest that Vapyrin-bodies have an identity that involves features of recycling endosomes and TGN, and that they are associated with the Golgi apparatus.

### Search for Interacting Partners of VAPYRIN

Since VAPYRIN has no predicted transmembrane or membrane association domain, and since both, the VAP domain and the ankyrin domain, are known to interact with integral membrane proteins in other eukaryotic systems, we reasoned that the localization of tagged VAPYRIN to Vapyrin-bodies may reflect interactions with resident membrane proteins. In order to identify such interacting partners of VAPYRIN we decided to perform co-immuno-precipitation (CoIP). Thus, VAPYRIN-GFP and VAP-GFP were expressed either transiently in *N. benthamiana*, or in the native context, namely in *P. hybrida* stably transformed by *Agrobacterium tumefaciens* through leaf disc transformation, and in hairy roots of *P. hybrida* transformed by *Agrobacterium rhizogenes* (see Supplementary Materials and Methods for more details).

In general, expression of VAPYRIN-GFP and VAP-GFP was always observed at low levels, although the constitutive cauliflower mosaic viral 35S-promoter was used. In addition to the low levels of expression, part of the fluorescent signal corresponded to free GFP (Figure S9). This suggests that VAPYRIN-GFP can be cleaved, and may reflect the cytoplasmic and nuclear signal in roots that express VAPYRIN-GFP (Figure 1). Due to the generally low expression level of VAPYRIN-GFP, and to the partial cleavage in the soluble fraction, we decided to search for interacting partners in the membrane-bound fraction. Hence, we attempted to solubilize VAPYRIN-GFP and VAP-GFP from purified microsomal membrane fractions (Figures S9–S12).

The central issue in solubilizing protein complexes for CoIP and MS analysis is to solubilize the complexes with detergents without interfering with the protein:protein interactions in the complex. A number of protocols have been established for receptors and other membrane proteins to achieve this goal (Avila et al., 2015). Using the non-ionic detergent Nonidet P-40 (NP-40) at a concentration of 1% (w/v), no VAPYRIN-GFP was solubilized from the membrane fraction (Figure S9), although this concentration is sufficient to solubilize the membrane receptor FLS2 (Chinchilla et al., 2006). Since both domains of VAPYRIN were localized to the same compartment, we reasoned that they may both interact with the same or adjacent target proteins in the same membrane, hence reinforcing the association with the target membrane. To avoid this problem, we next used only the N-terminal VAP domain of VAPYRIN fused to GFP to establish a solubilization protocol for CoIP. We prepared microsomal fractions from hairy roots transformed with VAP-GFP and treated them with different concentrations of three detergents that have been previously established for the solubilization of resident membrane proteins (le Maire et al., 2000; Arachea et al., 2012; Avila et al., 2015), namely NP-40, octylglucoside (OG), and Triton-X-100 (Figure S10). Since protein solubility can depend on pH, we also used different buffer systems (Figure S10). In addition, we used two protocols for the preparation of microsomal membranes, a classical protocol involving ultracentrifugation (Fabregas et al., 2013), and an alternative small-scale protocol that produces less dense membrane pellets, thereby facilitating solubilization (Abas and Luschnig, 2010). However, despite the use of up to 5% detergent, none of the protocols resulted in significant solubilization of VAP-GFP to amounts that would have allowed for CoIP (Figures S11, S12). These results document the strong association of VAPYRIN with the membrane of VAPYRIN-bodies.

### Yeast Two-Hybrid Screening for Interactors of VAPYRIN

Yeast two-hybrid interaction screens have been successfully used to identify protein-protein interactions in plants (Causier and Davies, 2002). We employed a system known as split-ubiquitin interaction screening that is based on the recognition of reconstituted ubiquitin resulting from the interaction of two hybrid proteins (Möckli et al., 2008). This system involves a membrane-bound bait at the ER surface. Because this interaction system operates outside the nucleus, it allows to use baits that have a tendency toward autoactivation. At the same time, this system is suited to identify protein-protein interactions that involve membrane proteins.

We first prepared a cDNA library derived from a 50:50 mixture of RNA from mycorrhizal and control roots. This library, which represented 4 × 10^6^ independent clones, was screened with the VAP domain as a bait (pDHB1-VAP). A total of 324 clones were recovered from a primary screen on 3.2 × 10^6^ clones (see Supplementary Materials and Methods for more details). After secondary screening for LacZ activity, 288 yeast clones were retained. From these candidate clones, plasmid DNA was extracted for amplification in *E. coli* and re-transformation of yeast for confirmation of the interaction with the VAP domain. Finally, 23 candidate clones were retained that showed a reproducible VAP-dependent growth phenotype in drop tests, and which were positive for ADE2 and LacZ activity (Figure S13; Table S3). Interestingly, 14 of the putative interactors (61%) were predicted to be membrane proteins with one to six predicted membrane-spanning domains.

**Figure 6:**
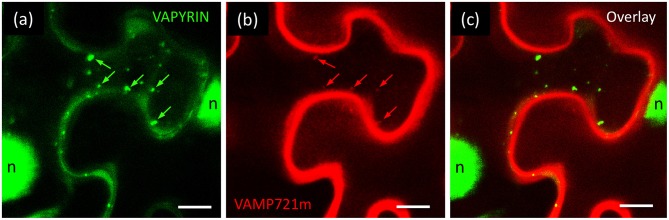
Co-localization of VAPYRIN with VAMP721m. Co-expression of VAPYRIN-GFP **(a)** with RFP-VAMP721m **(b)** revealed an overlapping localization pattern (arrows in **a,b**) **(c)**, consistent with an interaction of VAPYRIN with VAMP721m on the Vapyrin-bodies. Nuclear signal (*n*) was only observed in the green channel (reflecting cleaved VAPYRIN-GFP). Green arrows highlight Vapyrin-bodies, red arrows highlight the same objects with RFP-VAMP721m.

Interacting proteins are expected to colocalize on the same subcellular compartment. We therefore generated RFP fusions of the candidate interactors to test for subcellular colocalization with VAPYRIN-GFP. Only one candidate, which had been classified as a VAMP/R-SNARE protein by automated blast annotation (ID J17 I; Table S3) showed a significant overlap in localization with VAPYRIN-bodies (Figure 6). Because of its interaction with a mycorrhiza-related protein (VAPYRIN), it was designated as VAMP721m. In depth phylogenetic analysis showed that VAMP721m was closely related to VAMP721 in other species (Figure S14). Phylogenetic analysis of all VAMP72 members in *Petunia axillaris* (Pa), *M. truncatula* (Mt), *L. japonicus* (Lj), rice (*Oryza sativa*; Os), and *A. thaliana* (At) revealed that VAMP721m, together with 3 additional paralogs, (VAMP721x through VAMP721z) falls into a clade with the symbiotic VAMPs from *M. truncatula* (MtVAMP721d and MtVAMP721e) (Figure S14; File S1), that are required for AM (Ivanov et al., 2012). This clade does not contain a homolog of *Arabidopsis*, a phylogenomic signature that was found in other symbiosis-related genes (Delaux et al., 2014; Favre et al., 2014; Bravo et al., 2016). These results are compatible with the hypothesis that this clade contains symbiosis-related genes that have been under selection for a role in AM in the dicots.

Although Vapyrin-bodies exhibited RFP-VAMP721m signal (Figures 6a,b), an even stronger signal was observed along the cell periphery, possibly from the plasma membrane (Figures 6b,c). No RFP signal was observed in the nuclei, that usually exhibited GFP signal (Figures 1c, 3f, 6a) resulting from cleavage of VAPYRIN-GFP (Figure S9). These results suggest that the strong RFP-VAMP721m signal along the cell periphery does probably not reflect a cytoplasmic localization resulting from protein cleavage. In addition, these results exclude that the relatively weak RFP signal on Vapyrin-bodies could have resulted from “bleeding-through” from the GFP channel, which would also be the case for the nuclear signal.

It should be noted here that co-expression of VAPYRIN-GFP and RFP-VAMP721m in *N. benthamiana* proved rather difficult. In most cases, agro-infiltration experiments produced very few cells that co-expressed both markers, whereas this method worked routinely with other fluorescent markers (see above). These results suggest that the co-expression of VAPYRIN and VAMP721m at elevated levels is detrimental to tobacco leaf cells. As a consequence of the problems with co-expression and the resulting deficit in image quality, the imaging results from confocal microscopy of VAPYRIN-GFP and RFP-VAMP721m were not suitable for processing with the MotionTracker quantification pipeline.

More direct information on protein:protein interactions can be obtained with bi-molecular fluorescence complementation (BiFC). BiFC is based on the reconstitution of YFP fluorescence from two split halves that are brought together by the interaction of two fused proteins of interest (Kerppola, 2008). Using the system described in Waadt et al. (2008), we did not obtain a significant reconstitution of YFP fluorescence, with VAPYRIN and VAMP721m whereas positive controls yielded the expected readout (data not shown). This may be a consequence of the difficulty to co-express VAPYRIN and VAMP721m in the same cells (see above).

### Expression of VAMP Genes in Mycorrhizal Petunia

VAMPs, also known as R-SNAREs, are encoded by large gene families in angiosperms (Uemura et al., 2004; Sanderfoot, 2007). VAMP721m falls into the VAMP72 family, of which seven members were identified each in *A. thaliana* and *M. truncatula* (Uemura et al., 2004; Ivanov et al., 2012). Using VAMP721m as the query for a blastp search against the predicted *P. axillaris* proteome (Bombarely et al., 2016), we detected eight VAMP72 members (Figure S14) and four VAMP71 members, and we determined their gene expression patterns in mycorrhizal roots vs. control roots from a recently generated RNAseq data set (Rich et al., 2017a) (Table S4). Interestingly, none of the 12 petunia VAMP72 and VAMP71 members were significantly regulated upon mycorrhizal colonization, except for two that were slightly but significantly repressed in mycorrhizal roots (Peaxi162Scf00149g01112.1, Peaxi162Scf00367g00010.1). Notably, VAMP721m was not induced during AM symbiosis (Table S4). This is reminiscent of the symbiosis-related VAMP721 homologs in *M. truncatula, VAMP721d* and *VAMP721e*, the expression of which remained constant in AM and during nodulation (Ivanov et al., 2012).

## Discussion

### Vapyrin-Bodies Can Be Formed in Diverse Plant Species and Cell Types

In order to study the identity and function of Vapyrin-bodies, we tested two expression systems with VAPYRIN-GFP: transgenic *Arabidopsis* roots, and agro-infiltrated tobacco leaves (*N. benthamiana*). Both exhibited distinct Vapyrin-bodies, however, in *Arabidopsis* roots, they were immobile, suggesting that *Arabidopsis* has subcellular compartments similar to Vapyrin-bodies, but lacks a trafficking component required for their active translocation. In contrast, tobacco leaves exhibited Vapyrin-bodies with a similar behavior as in *P. hybrida* roots (Feddermann et al., 2010) or *M. truncatula* roots (Pumplin et al., 2010). Hence, we further employed agro-infiltrated tobacco leaves for the characterization of Vapyrin-bodies.

### Both Domains of VAPYRIN Bind to VAPYRIN-Bodies

Usually, subcellular localization of proteins is determined by specific peptide signatures in the primary amino acid sequence. In VAPYRIN, no such sequence was found that would have allowed to predict its subcellular localization. Hence, it was surprising to find that both, the VAP domain and the ANK domain, independently mediated localization to the same endosomal compartment (Figures 2a,b, 4). This shows that both domains together contribute to VAPYRIN localization, and it explains the fact that VAPYRIN is very strongly bound to membranes (Figures S9–S12). VAP domains can be expected to localize to the membranes of vesicles through their interaction with VAMPs (Lev et al., 2008). Similarly, the ANK domain is known as the membrane-binding domain of ANKYRIN proteins in animals (Michaely et al., 2002; Wang et al., 2014). In particular, some conserved regions on the inner (concave) side of the solenoid structure of the crescent-shaped ANK domain represent a strong interaction surface, with which it interacts with resident membrane proteins such as ion channels (Wang et al., 2014). Importantly, such binding areas are usually constituted by several adjacent ankyrin repeats (Cunha and Mohler, 2009), and indeed, VAPYRIN carries a similar highly conserved region of several ankyrin repeats on the concave side of the ANK domain, indicating that this region may represent a conserved interaction surface (Feddermann and Reinhardt, 2011).

### Movement of VAPYRIN-Bodies

Vapyrin-bodies move rapidly in plant cells (Feddermann et al., 2010; Pumplin et al., 2010), indicating that they may play a role in transport and/or secretion of either membrane material or an unknown cargo. Since VAPYRIN-bodies are often observed in the vicinity of the fungal tips in cells with developing arbuscules, they have been implicated in the delivery of factors required for fungal morphogenesis and growth (Zhang et al., 2015). Vapyrin-bodies do not exhibit a steady mode of movement, but they either move rapidly along ER strands (Figure S5), or they suddenly stop and pause at particular sites, before moving further in the same or in another direction. This pattern of movement has been described as stop-and-go movement, which is characteristic for the movement of subcellular compartments such as Golgi stacks (Boevink et al., 1998; Nebenführ et al., 1999). Recently, the mechanistic basis of this behavior has been proposed to represent an ER-based, actin-driven transport system that interacts with microtubules at specific sites referred to as “cortical microtubule-associated endoplasmic reticulum sites” (C-MERs) (Pena and Heinlein, 2013). This movement pattern is fundamentally different from the general movement of the cytoplasmic constituents, known as cytoplasmic streaming.

### Quantitative Image Analysis Reveals Endosomal Identity of Vapyrin-Bodies and Association With the Golgi Apparatus

**Figure 7:**
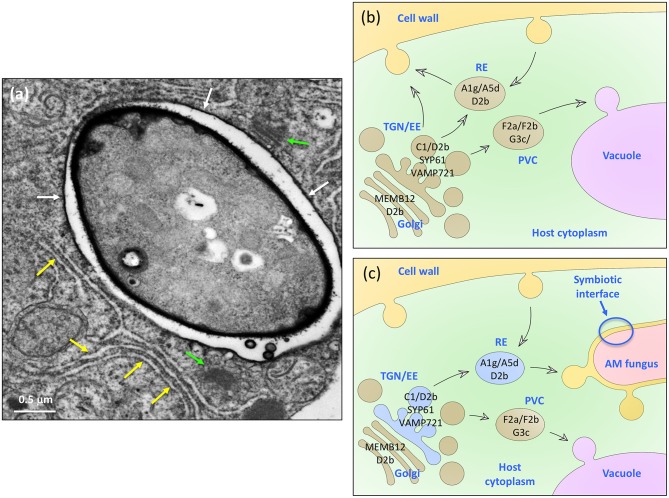
A model for the role of VAPYRIN and the Vapyrin-bodies in AM symbiosis. **(a)** Transmission electron micrograph of an AM fungal hypha in a colonized petunia root cortex cell. The hypha is surrounded by a peri-arbuscular membrane (white arrows) and embedded in an organelle-rich cytoplasmic pocket with Golgi stacks (green arrows), and numerous ER cisternae (yellow arrows). Size bar: 0.5 μm. **(b)** Schematic representation of the constitutive trafficking system in a non-colonized plant cell with subcellular compartments such as Golgi stacks, trans-Golgi network (TGN) and early endosomes (EE), recycling endosomes (RE), and the prevacuolar compartment (PVC). Protein markers used in this study are indicated in black on the respective organelle, to which they localize. **(c)** Schematic representation of a mycorrhizal cell with the analogous complement of subcellular trafficking intermediates as in **(b)**. In addition, VAPYRIN is indicated as blue color in the compartments that were identified in our study by co-localization with subcellular markers. Note that the default secretion pathway is diverted toward the AM fungus.

In addition to visual readouts (Figures 1–3), we performed a quantitative analysis of protein co-localization (Figure 4), and organelle association (Figure 5). This allowed us to reliably characterize the Vapyrin-bodies relative to well-defined markers for subcellular compartments in plants (Geldner et al., 2009). With this approach, we excluded an identity of vacuolar, prevacuolar, late endosomal, mitochondrial, and peroxisomal identity. Instead, we show that Vapyrin-bodies have an endosomal identity. Using a range of endosomal markers which reveal specific subpopulations of the different endosomal compartments, we then narrowed down the identity of the Vapyrin-bodies. In particular, the endosomal/TGN markers RabC1, RabD2b, SYP61 and the (recycling) endosome marker RabA5d co-localized with Vapyrin-GFP (Figures 2, 4). These results indicate that Vapyrin-bodies have a trans-Golgi/endosomal identity with an additional component of recycling endosomes (Figure 7). This rather broad identity may explain the fact that the size of Vapyrin-bodies is variable (Figures 1–3), reflecting either a certain heterogeneity in actual size of the compartment, or the aggregation of multiple closely associated compartments that cannot be resolved by confocal microscopy.

The fact that none of the late endosomal/prevacuolar/vacuolar markers overlapped with VAPYRIN-GFP suggests that VAPYRIN is not involved in trafficking toward the vacuole. Instead, it appears that Vapyrin-bodies may be involved in anterograde trafficking from the Golgi. Considering the fact that colonized mycorrhizal cells exhibit a redirected default transport route toward the symbiotic interface (Pumplin et al., 2012) we hypothesize that VAPYRIN-bodies are involved in transport from the Golgi to arbuscular branches (Zhang et al., 2015).

### VAPYRIN Interacts and Co-localizes With a Close Homolog of the Symbiotic R-SNAREs MtVAMP721d and MtVAMP721e

In order to identify interacting partners of VAPYRIN, we carried out a yeast-two-hybrid (Y2H) screen with the VAP-domain as a bait. With this strategy, we identified a vesicle-associated membrane protein (VAMP721m) that co-localized with VAPYRIN (Figure 6). Phylogenetic analysis identified it as a close homolog of the symbiotic VAMPs in *M. truncatula* VAM721d and VAMP721e (Figure S14), which fall into a separate symbiosis-related clade (Ivanov et al., 2012). As in the case of *VAM721d* and *VAMP721e*, the *VAMP721m* gene exhibits constitutive expression and does not respond to AM colonization (Table S4). This is consistent with the idea that VAPYRIN is a symbiosis-specific component that interacts with a constitutive and ubiquitous secretion pathway that is not restricted to symbiotic organs or to plant species competent to engage in endosymbiosis. In this sense, VAPYRIN would represent a symbiosis-related component of a pathway that interacts with a constitutive secretion machinery through VAMP721m. Hence, we hypothesize that the redirection of the secretory route toward the fungus in colonized cells (Pumplin et al., 2012) may require only a few symbiosis-specific components such as VAPYRIN and EXO70I (Pumplin et al., 2012; Zhang et al., 2015), or SYP132 that has overlapping functions in symbiosis, and defense (Catalano et al., 2007; Kalde et al., 2007; Huisman et al., 2016; Pan et al., 2016).

### Are VAPYRIN-Bodies Involved in Secretion?

Taken together, our results complement the findings that VAPYRIN interacts with EXO70I (Zhang et al., 2015), a component of the exocyst complex, which promotes exocytosis and secretion into the apoplast (Zhang et al., 2010). In colonized cells of *M. truncatula*, VAPYRIN and EXO70I have been shown to be located in the vicinity of the peri-arbuscular membrane (PAM) around the tips of branching hyphae (Zhang et al., 2015). These are sites of intense exocytotic activity (Genre et al., 2012). Interestingly, the secretion of the AM-associated ABC transporters STR and STR2 is abnormal in *exo70i* mutants of *M. truncatula*, consistent with the idea that VAPYRIN-bodies are involved in secretion of membrane constituents of the PAM. Symbiotic cells need an active secretion pathway, in order to deliver to the symbiotic interface nutrient transporters (Pumplin et al., 2012), extracellular proteases (Takeda et al., 2007), and possibly other cargo that is essential for AM fungi. A role for VAPYRIN-bodies in secretion is not in contradiction with their endosomal identity, since endosomes can be integrated as recycling endosomes into the anterograde secretory pathway from the trans-Golgi to target membranes (Figure 7) (Robinson et al., 2008). In this context, it is interesting to note that the membrane dynamics in symbiotic cells involves markers of both, endocytosis and secretion, thus indicating the operation of active endosomal recycling in mycorrhizal cells (Russo et al., 2019).

### A Model for the Function of VAPYRIN in Symbiosis

Mycorrhizal cells undergo fundamental reorganization, starting from installation of the PPA (Genre et al., 2005, 2008). Subsequently, colonized cells induce hundreds of AM-related genes, and finally, the symbiotic interface has to be established for bidirectional nutrient transfer (Karandashov and Bucher, 2005; Rich et al., 2017b; Roth and Paszkowski, 2017). This involves increased secretion activity (Genre et al., 2012), and redirection of secretion toward the symbiotic interface (Pumplin et al., 2012). Arbuscular fine hyphae are surrounded by large amounts of cytoplasm rich in organellar constituents (Figure 7a). Based on the collective available evidence, it appears that plant cells normally have a secretion pathway with two main directions, a route to the plasma membrane and one to the vacuole (Figure 7b). In colonized cells, transport is redirected to the fungus, involving an endosomal compartment that carries VAPYRIN and VAMP721 (Vapyrin-bodies). This endosomal compartment has both TGN and RE identity, and is as such not restricted to specific cell types or AM-competent plant species. Upon mycorrhizal colonization of root cells, these compartments carry VAPYRIN and become recruited for symbiosis-related functions, possibly secretion of membrane material and/or cargo to the symbiotic interface at the tips of arbuscular fine branches (Figure 7C).

## Experimental Procedures

### Plant Material

Seeds of *Nicotiana benthamiana* and *Petunia hybrida* were surface sterilized in 70% ethanol (1 min), followed by 7% bleach with 0.1% Tween 20, and rinsed 5 times before sawing to seedling substrate (Klasman). After 1 week from germination, plantlets were transferred to single pots with clay substrate (Klasman). Once a week soil was treated with iron fertilizer (Optifer from Optima). Temperatures in growth chambers were adjusted to 32°C during the light phase (12 h) and 22°C during the dark phase (12 h). For the production of sterile *in vitro* plant material for transformation (see Supplementary Materials and Methods), sterilized seeds were plated onto MS medium.

### Transient Expression by Agro-Infiltration and Confocal Microscopy

Transient expression assays were performed with three to 5-week-old plants as described (Leuzinger et al., 2013) with some modifications. Briefly, *A. tumefaciens* strains transformed with the vector of interest were grown for 2 days at 28°C on solid LB media with the appropriate antibiotics. One colony was picked and grown overnight in 3 ml LB medium with the appropriate antibiotics at 28°C and 210 rpm. The pre-culture was then diluted 1:1000 in 15–20 ml LB with the appropriate antibiotics and incubated overnight at 28°C and rotation (210 rpm). The culture was then centrifuged at 4,000 g for 15 min at room temperature. The pellet was resuspended and adjusted to an OD600 of 0.7 in AS medium (For 100 ml: 1 ml 1 M MES-KOH pH 5.6, 333 μl 3 M MgCl_2_ and 100 μl 200μM acetosyringon). Each culture was mixed 1:1 with a culture expressing the viral repressor p19 (Voinnet et al., 2003), or 1:1:1 in the case of co-expression with to fluorescent proteins. The cultures were incubated at room temperature for 2–4 h under gentle shaking. The leaf abaxial side of 2–3 *N. benthamiana* plants per construct were infiltrated with bacterial suspensions with a 1 ml syringe. The infiltrated leaves were analyzed after 2–5 days by confocal laser scanning microscopy with a Leica SP5 microscope. For eGFP and YFP, an argon laser was used for excitation (488 nm), and signal was acquired between 500 and 550 nm. For mCherry and mRFP, a helium-neon laser was employed for excitation (543 nm), and fluorescence was acquired between 590 and 640 nm. Confocal images were analyzed with the Leica LAS AF Lite and FIJI/ImageJ softwares. All double-channel analyses were acquired in the sequential mode to avoid “bleeding-through” between the channels. For the analysis of the dynamic behavior of Vapyrin-bodies, and for quantitative analysis of colocalization and association (see below), time series (movies) were acquired. For each construct, at least three independent transformation experiments were conducted, each with several plants of which several leaves were infiltrated. Representative movies were chosen for quantitative analysis.

### Quantitative Analysis of Co-localization and Association

Quantification of object co-localization and association was performed with a semi-automated image processing pipeline based on the Kalaimoscope MotionTracker software version 8.88.15 (Kalaidzidis et al., 1997; Rink et al., 2005; Collinet et al., 2010), running in Windows 10 (64 bits) (http://www.kalaimoscope.com/science.html). This software identifies elliptic objects by fitting Lorenzian functions to them according to user-defined object-search parameters. This allows to determine the x-y position with sub-pixel accuracy, as well as further object properties including size, area, intensity and position. Once the object search is complete for all the consecutive frames of a time-series (movie) in two channels, MotionTracker can then search for tracks. To this end, its algorithm searches the most probable association between objects in adjacent frames of the two channels, and establishes the path of all objects during the time-series. Object size, speed and direction are calculated to determine the object tracks. The visualization of the object tracks by plotting their path as an overlay on the image sequence allows easy visual inspection of the object tracking results to confirm their accuracy and, if necessary, to adjust the tracks manually.

To extract the co-localization data, MotionTracker computes the overlap between objects by determining the peak cross-section of the fitted Lorenzian at half-height and by computing the area that is covered by another cross-sections belonging to a different channel. The co-localization measure was parametrized by a co-localization threshold set at 50% overlap (Priya et al., 2015), i.e., each object in channel A overlapping with an object in channel B with at least half of its area was counted as a co-localized object. Finally, the co-localization data plotted in Figure 4 represent the percentage of all the objects of a movie fulfilling the above criteria. Since co-localization is asymmetric in cases where object size and/or expression level differ between the two channels, these calculations were performed with both GFP and RFP as the basis channel.

Object association was quantified as the distance between two objects in the red and the green channel, respectively, over time. For this analysis, the tracks of associated pairs of objects were first identified by their distance of ≤2 μm between their centers of mass, over at least 9 consecutive frames (average 21.7 frames) (Figures 5a–c, left). With an average object size of ca. 0.65 μm, this distance threshold includes objects that are either in close proximity, touching each other, or overlapping. This first filtering allowed to identify pairs of objects that appeared to be physically connected. For two markers that associated with Vapyrin-bodies (24R and 33R), and a marker that was shown to colocalize (3R), the distance was then measured for at least 5 paired tracks. Distances were then plotted over time for all the analyzed tracks (Figures 5a–c, middle). The same data was then binned to distance classes to show the distribution of distances of the entire analyzed data set (Figures 5a–c, right).

### Yeast 2-Hybrid Screening Procedures and Bait Cloning

In order to identify interacting partners of VAPYRIN, the split-ubiquitin Y2H screen from DUALhunter was employed (DUALsystems Biotech AG, Switzerland). All procedures were carried out as detailed in the manufacturers protocol (). To prepare the bait vector, the VAP domain was amplified by PCR from cDNA prepared from RNA extracted from mycorrhizal petunia roots using the following primers:

Forward primer:

5′-GAGTGGCCATTACGGCCATGGATAGACTATTAAGCTTAGAGCCATC-3′; Reverse primer:

5′-TCGACATGGCCGAGGCGGCCGTAGCTCCAGGGGCTACAAC-3′.

Both primers contained asymmetric SfiI restriction sites to allow directional cloning into the SfiI sites of the pDHB1 vector. The amplicon was cloned into pGEM®T-easy (Promega, USA), sequenced and recovered by SfiI digestion (New England Biolabs, Inc.:U.S.). It was then ligated into pDHB1 to obtain the pDHB1-VAP bait vector and transformed into *Saccharomyces cerevisiae* reporter strain NMY51 (kindly provided by Claudio de Virgilio) by electroporation according to the manufacturers protocol. *S. cerevisiae* NMY51 is auxotrophic for tryptophan, leucine, histidine, and adenine [MATa his3Δ200 trp1-901 leu2-3,112 ade2 LYS2::(lexAop)_4_-HIS3 ura3::(lexAop)_8_-lacZ ade2::(lexAop)_8_-ADE2 GAL4]. All procedures were carried out according to the recommendations of the provider.

### Preparation of a Prey Library for Split Ubiquitin Screening

The cDNA library (prey vectors) was constructed with the EasyClone cDNA Library Construction Kit (Dualsystems Biotech No. P01010), according to the provider's recommendations. In brief, 5 μg total RNA extracted from a 50:50 mixture of non-colonized roots and mycorrhizal roots colonized by *R. irregularis* MUCL 43,204 was used for cDNA synthesis. Before reverse transcription, mRNA was enriched with Oligotex Direct mRNA Mini Kit (Qiagen, Germany). The first strand cDNA was synthesized by the EasyClone reverse transcriptase (RT) using a 3′-end adapter containing an SfiI site and oligo (dT). This RT reaction adds oligo (dC)s to the 5′-end of the first strand, allowing the 5′-end adapter that contains the second SfiI site to bind. An evaluative PCR using 1 μl of the first strand cDNA was performed to determine the optimal number of cycles for PCR amplification. Based on this test, the second strand was amplified by PCR from the first strand cDNA with 17 cycles, using primers containing SfiI sites A and B. DNAs were purified with the NucleoSpin Extract II kit (Macherey-Nagel, Germany) and digested with 2 μl SfiI restriction enzyme during an overnight incubation at 50°C. cDNAs longer than 400 bp were selected by size fractionation (CHROMA SPINTM-400 columns; Clontech). Note that in contradiction to the recommendations of the provider, cDNAs were collected in the third elution, which was controlled by gel electrophoresis. cDNAs were purified by phenol/chloroform extraction and quantified using a NanoDrop spectrophotometer (Thermo Fisher Scientific). The cDNA was then directionally inserted downstream of NubG using two asymmetric SfiI restriction sites. A test transformation in *E. coli* Top10 was performed to check for the number of independent transformants in the final library. To test the quality of the library, 10 randomly picked colonies were analyzed by colony PCR with pPR3N primers.

### Optimization of Screening Conditions

To optimize the stringency of the screen, a first pilot screen was carried out that simulated the conditions of a library screen, but with the empty library vector (no bait) instead of the cDNA library vectors. NMY51 cells expressing DHB1-VAP were pre-cultured in liquid SD-Leu, then grown to an OD600 of 0.6–0.8 in 2x YPAD, and transformed with pPR3-N (empty library vector; EV) using a high-efficiency library scale transformation. For that, they were incubated 45 min. at 30°C with the master mix and the plasmids and then heatshocked for 20 min. at 42°C with DMSO. Transformed yeast was re-suspended in 2x YPAD and incubated 90 min. at 30°C with slow shaking for recovery. The cells were then resuspended in 0.9% NaCl and plated on different media of increasing stringency: (i) SD-2D: three 100 mm ø plates, to test for transformation efficiency. (ii) SD-3D: six 150 mm ø plates lacking W, L and H, each supplemented with an increasing concentration of 3-aminotriazole (3-AT): 0, 1, 2.5, 5, 7.5, 10 mM. (iii) SD-4D: six 150 mm plates lacking W, L, H, and A, containing the same concentration of 3-AT as SD-3D plates. Plates were then incubated at 30°C and yeast growth was assessed. VAP bait should not interact with the empty library vector (Table S3). However, the HIS3 reporter gene can be leaky and lead to unspecific growth. To inhibit this background growth and increase the selection stringency, 3-AT, a competitive inhibitor of the HIS3 gene product is used. SD-4D plates are more stringent that SD-3D. Since too stringent conditions lead to false negative and not stringent enough conditions leads to false positive, the pilot screen was performed to determine the optimal medium to use for the library screen with pDHB1-VAP. This medium corresponds to the plates where no background growth was observed with the minimal amount of 3-AT.

### Isolation and Characterization of VAP Interactors

VAP-expressing NMY51 was transformed with 28 μl library plasmid with a high-efficiency transformation method (see above). Transformants were plated on SD-2D plates to test for transformation efficiency and on SD-4D without 3-AT, because this condition was establishes as 100% selective. Positive clones where recovered and cultured on SD-2D plates for the X-Gal filter assay. For further characterization, DNA from yeast clones with putative interactors was extracted and transformed into XL1-blue. Plasmid DNA from transformed bacteria was extracted with ZR miniprep kit and tested for candidate cDNA insert by digestion with SfiI.

Interaction strength and specificity with the VAP domain was determined by a drop test. Candidate clones were re-transformed into NMY51expressing the bait VAP fusion protein. Transformed yeast was cultured in 2 ml selective medium (SD-2D) overnight at 30°C, adjusted to an OD600 of 0.2 and re-grow to an OD600 of 0.8–3. 200 μl of each culture was diluted to an OD600 of 1 in a microtiterplate in 4 dilution series: 1/1, 1/10, 1/100, 1/1000. Sterile water was used for the dilutions. 3 μl of each dilution were spotted onto 2 plates in parallel: SD-2D, to test for the presence of the plasmids and the cell number of a drop and SD-4D, to test for protein-protein interaction of the bait and prey protein pairs. After 24 h at 30°C, colonies started to be visible on SD-2D plates. After 36–48 h, growth was observed on SD-4D plates. Pictures were taken after 36 h for SD-2D plates and after 3 days for SD-4D plates. Control vectors were used for comparison in combination with pDHB1-VAP; pA1-Alg4 as a positive control and pPR3-N (EV) as a negative control of interaction. Additionally, each candidate clone was also tested with the empty bait vector (pDHB1, EV) by a drop test to test for specificity of the interaction with the VAP domain. Prey candidates interacting strongly with the empty bait were eliminated, the other candidates were considered true interactors of VAP. Additional information concerning the biochemical and genetic search for interactors of VAPYRIN can be found in the Supplementary Materials and Methods (File S2).

### Phylogenetic Analysis

Sequence comparison and generation of the phylogenetic tree with VAMP721m and closely related family members (File S1) was carried out with the predicted amino acid sequences using the “advanced” function of the software package at www.phylogeny.fr (Dereeper et al., 2008), with 100 bootstrap replicates.

## Short Summary

VAPYRIN interacts with VAMP721 on an endosomal compartment (Vapyrin-bodies) required for the establishment of arbuscular mycorrhiza.

## Data Availability

All datasets generated for this study are included in the manuscript and/or the Supplementary Files.

## Author Contributions

LB, SL, GD, CM, NF, and MC conducted experiments. GD and FM analyzed microscopic data. DR conceived the project and wrote the manuscript with the help of all authors.

### Conflict of Interest Statement

The authors declare that the research was conducted in the absence of any commercial or financial relationships that could be construed as a potential conflict of interest.

## Supplementary Material

Data Sheet 1 contains Figures S1 to S8; Data Sheet 2 contains Figures S9 to S14 and Tables S1 to S4; Data Sheet 3 contains File S1; Data Sheet 4 contains File S2 with Supplementary Materials & Methods.

Figure S1 Subcellular localization of VAPYRIN protein domains VAP and ANK. Simultaneous co-expression of full-length VAPYRIN tagged with RFP (VAPYRIN-RFP) together with GFP-tagged VAP **(a)** and ANK **(b)** domains reveals complete co-localization on Vapyrin-bodies. Positive control: VAPYRIN-GFP together with VAPYRIN-RFP **(c)**. Size bar: 10 μm.

Figure S2 Co-localization of VAPYRIN with endosomal markers. Co-expression of VAPYRIN-GFP with the endosomal marker mCherry-RabD2b **(a)**, with the trans-Golgi/endosomal marker mCherry-RabC1 **(b)**, and with the Golgi marker mCherry-MEMB12 **(c)**. Size bar: 10 μm.

Figure S3 Association of Vapyrin-bodies with Golgi stacks. A consecutive series of images from a movie reveals the long-term association of Vapyrin-bodies with MEMB12-compartments. Time steps are 5.16 seconds, a white circle highlights a small group of paired compartments. Size bar: 10 μm.

Figure S4 Co-localization of VAPYRIN with markers for post-Golgi and recycling endosome. Co-expression of VAPYRIN-GFP with the post-Golgi marker mCherry-SYP61 **(a)**, with the trans-Golgi/endosomal marker mCherry-RabA5d **(b)**, and with the endosomal/recycling endosome marker mCherry-RabA1g **(c)**. Size bar: 10 μm.

Figure S5 Association of Vapyrin-bodies with the ER system. A series of images from a movie reveals the association of Vapyrin-bodies (arrows; marked with VAPYRIN-RFP) with the endoplasmic reticulum (marked with GFP-HDEL), and their movement along ER strands (orange arrow). Time steps are indicated. Size bar: 5 μm.

Figure S6 Co-expression of VAPYRIN with pre-vacuolar markers. Co-expression of VAPYRIN-GFP with the late endosomal/prevacuolar marker mCherry-RabF2a **(a)**, with the late endosomal/pre-vacuolar marker mCherry-RabF2b **(b)**, and with the late endosomal/vacuolar marker mCherry-RabG3c **(c)**. Size bars: 10 μm.

Figure S7 Co-expression of VAPYRIN with non-endosomal markers. Co-expression of VAPYRIN-GFP with the mitochondrial marker pIVD145seqFP611 **(a)**, with the peroxisomal marker GFP(S65T)-APX(36) **(b)**, and with the plasma membrane marker RFP-PIP1;4 **(c)**. Size bar: 10 μm.

Figure S8 Concept of quantification of colocalization. **(a)** Intensity plot of an image with two adjacent nearly circular objects in the green and red channel, respectively. At the half intensity level (blue arrow) the objects are hardly touching. **(b)** Intensity plot as in **(a)** with two objects that are virtually overlapping. **(c)** Definition of the threshold level for co-localization. Objects were considered to co-localize if their cross-section at half-height intensity overlapped by at least 50%.

Figure S9 Preparation of microsomal membranes and attempted solubilization of VAPYRIN-GFP. **(a)** Western blot with monoclonal anti-GFP antibody using *N. benthamiana* leaves expressing VAPYRIN-GFP. After removal of crude cellular debris, the supernatant (input) was ultracentrifuged to yield microsomal membranes (MEM) and soluble supernatant (SP). The MEM fraction was then treated with 1% NP-40 in order to solubilize VAPYRIN-GFP, but it remained entirely associated with the MEM fraction. **(b)** Same procedure as in **(a)**, but with leaves expressing ER-localized GFP-HDEL. **(c)** Same procedure as in **(a)**, but with leaves expressing free cytoplasmic GFP.

Figure S10 Experimental conditions in tissue fractionation for the extraction and solubilization of VAPYRIN-GFP and VAP-GFP. Transgenic hairy roots of *P. hybrida* were used to extract membrane pellets with a large-scale and a small-scale protocol (see Materials and Methods). Subsequently, the membranes were resuspended in different buffers containing various concentrations of different detergents as indicated.

Figure S11 Attempted solubilization of VAP-GFP with NP-40. Microsomal membrane fractions prepared from hairy roots expressing VAP-GFP were resuspended in different buffers with various concentrations of NP-40 as indicated. After incubation for 1 h at 4°C, the membrane pellets (MEM) were recovered by centrifugation and subjected to Western blot analysis next to the respective soluble fraction (SP; supernatant after detergent treatment). In all cases, most of the VAP-GFP remained associated with the membranes.

Figure S12 Attempted solubilization of VAP-GFP with Triton-X-100 and octylglucoside. Microsomal membrane fractions prepared from hairy roots expressing VAP-GFP were resuspended in different buffers with various concentrations of Triton-X-100 (T), or octylglucoside (OG) as indicated. After incubation for 1 h at 4°C, the membrane pellets (MEM) were recovered by centrifugation and subjected to Western blot analysis next to the respective soluble fraction (SP; supernatant after detergent treatment). In all cases, most of the VAP-GFP remained associated with the membranes.

Figure S13 Drop test with interactor candidates from split-ubiquitin screen. Interaction between the bait and the respective candidates is revealed by the white color and the better growth on selective medium (SD-4D) with the bait (pDHB1-VAP), relative to the empty vector control (pDHB1-EV). Growth on non-selective medium (SD-2D) is independent of an interaction and just reflects the presence of the bait and prey vectors. Highly specific interactors are signified by a + sign, moderately specific interactors are indicated with a ~ sign. A positive control is provided by pA1-Alg5 vs. pPR3N.

Figure S14 Phylogenetic analysis of petunia VAMP721. The petunia VAMP721m (red asterisk) in this study was compared with all related VAMPs from *Petunia axillaris* (Pa), *Medicago truncatula* (Mt), *Lotus japonicus* (Lj), *Oryza sativa* (Os), and *Arabidopsis thaliana* (At). Symbiosis-related (blue asterisks), and non-symbiotic (black asterisks) VAMP721 members from *M. truncatula* are indicated.

Table S1 List of subcellular fluorescent makers employed in this study.

Table S2 Statistical analysis of quantitative co-localization analysis. Tukey HSD *post-hoc* test was performed to test for the significance of co-localization (see Figure 4). Two clear groups emerged: ANK-GFP, VAP-GFP, VAPYRIN-RFP, RabD2b-RFP, RabC1-RFP, and RabA5d-RFP significantly co-localized with VAPYRIN-RFP or VAPYRIN-GFP, respectively, whereas MEMB12, RabA1g, RabF2a, and eqFP611 did not. SYP61 took an intermediate position, but was closer to the colocalizing group.

Table S3 List of interactor candidates identified by the split-ubiquitin screen. Indicated are assigned function by automatic BLAST, recovered protein length, % recovered protein relative to the full-length protein, presence of a signal peptide (cleavage site indicated), predicted subcellular localization, and the number of predicted transmembrane TM domains in the entire protein.

Table S4 RNAseq data for 12 VAMP genes of *P. hybrida* in mycorrhizal roots vs. control roots. Indicated are the individual RPKM values for mycorrhizal samples (M1–M3), and control samples (c1–c3), as well as the respective averages (M ave and c ave, respectively). Asterisks indicate *p*-values from Baggerley's test for differences between treatments M and c (original values—FDR *p*-value correction), and the induction ratio mycorrhizal vs. control (M/c) (see Rich et al., 2017a for further information).

Movie S1 Expression of VAPYRIN-GFP by agro-infiltration in *N. benthamiana*.

Movie S2 Expression of VAPYRIN-GFP in stably transformed *A. thaliana*.

Movie S3 Co-expression of VAPYRIN-GFP with mCherry-RabD2b by agro-infiltration in *N. benthamiana*.

Movie S4 Co-expression of VAPYRIN-GFP with mCherry-RabC1 by agro-infiltration in *N. benthamiana*.

Movie S5 Co-expression of VAPYRIN-GFP with mCherry-MEMB12 by agro-infiltration in *N. benthamiana*.

Movie S6 Co-expression of VAPYRIN-GFP with mCherry-RabA5d by agro-infiltration in *N. benthamiana*.

File S1 Protein sequences used for phylogenetic analysis of VAMP proteins.

## Abbreviations

ANK: Ankyrin domain
AM: Arbuscular mycorrhiza
ER: Endoplasmic reticulum
PAM: Peri-arbuscular membrane
PPA: Pre-penetration apparatus
RE: Recycling endosome
RNS: Root nodule symbiosis
TGN: Trans-Golgi network
VAMP: Vesicle-associated membrane protein
VAP: VAMP-associated protein.
