# Supplementary material for: VAPYRIN Marks an Endosomal Trafficking Compartment Involved in Arbuscular Mycorrhizal Symbiosis
Source: Front Plant Sci. 2019 Jun 4;10:666. doi: 10.3389/fpls.2019.00666 (PMC6558636; doi:10.3389/fpls.2019.00666)
Supplement: Figure S1 — Subcellular localization of VAPYRIN protein domains VAP and ANK. Simultaneous co-expression of full-length VAPYRIN tagged with RFP (VAPYRIN-RFP) together with GFP-tagged VAP (a) and ANK (b) domains reveals complete co-localization on Vapyrin-bodies. Positive control: VAPYRIN-GFP together with VAPYRIN-RFP (c). Size bar: 10 μm. [file Data_Sheet_1.pdf]

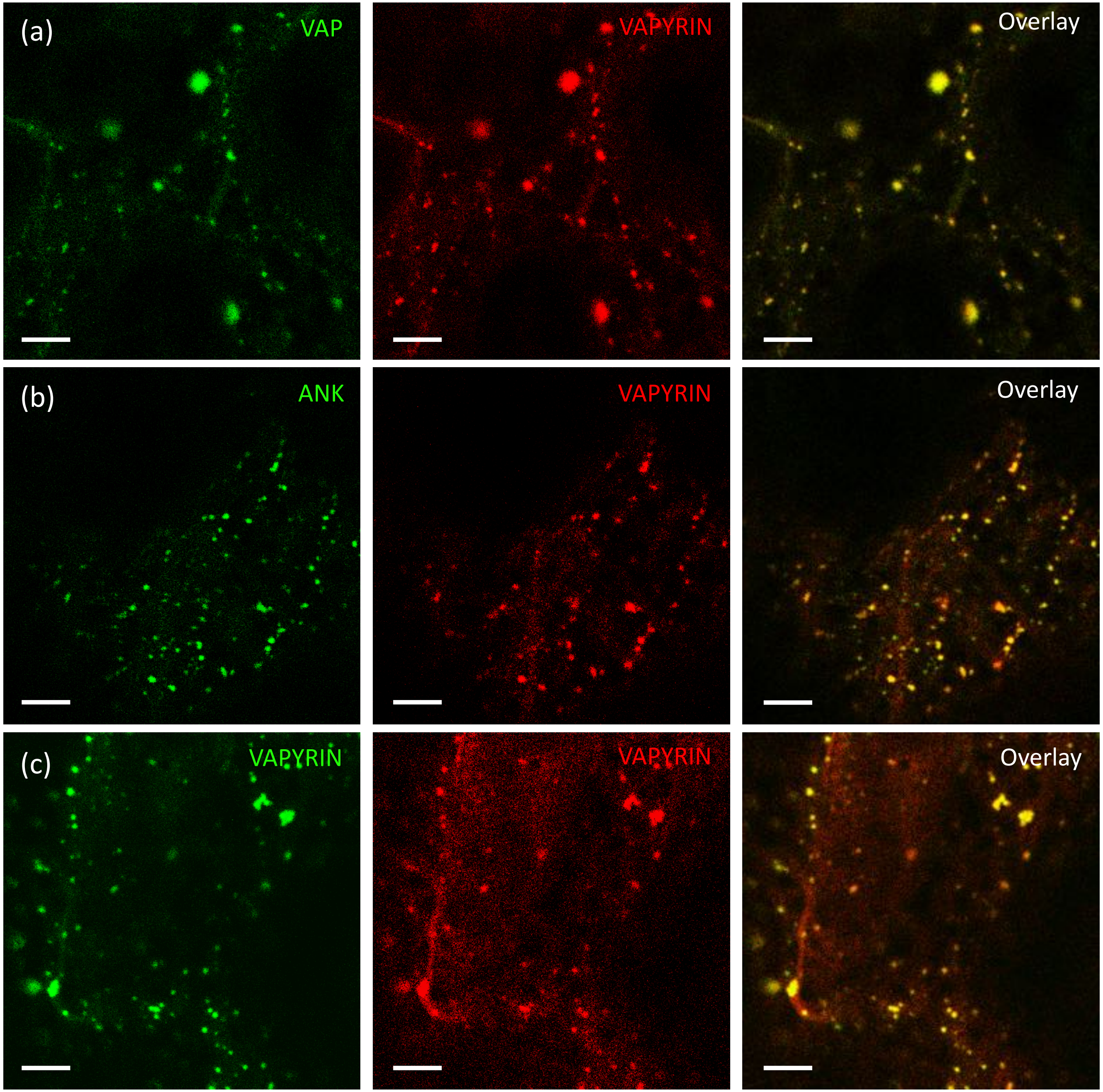

Figure S1 | Subcellular localization of VAPYRIN protein domains VAP and ANK. Simultaneous co-expression of full-length VAPYRIN tagged with RFP (VAPYRIN-RFP) together with GFP-tagged VAP (a) and ANK (b) domains reveals complete co-localization on Vapyrin-bodies. Positive control: VAPYRIN-GFP together with VAPYRIN-RFP (c). Size bar: 10µm.

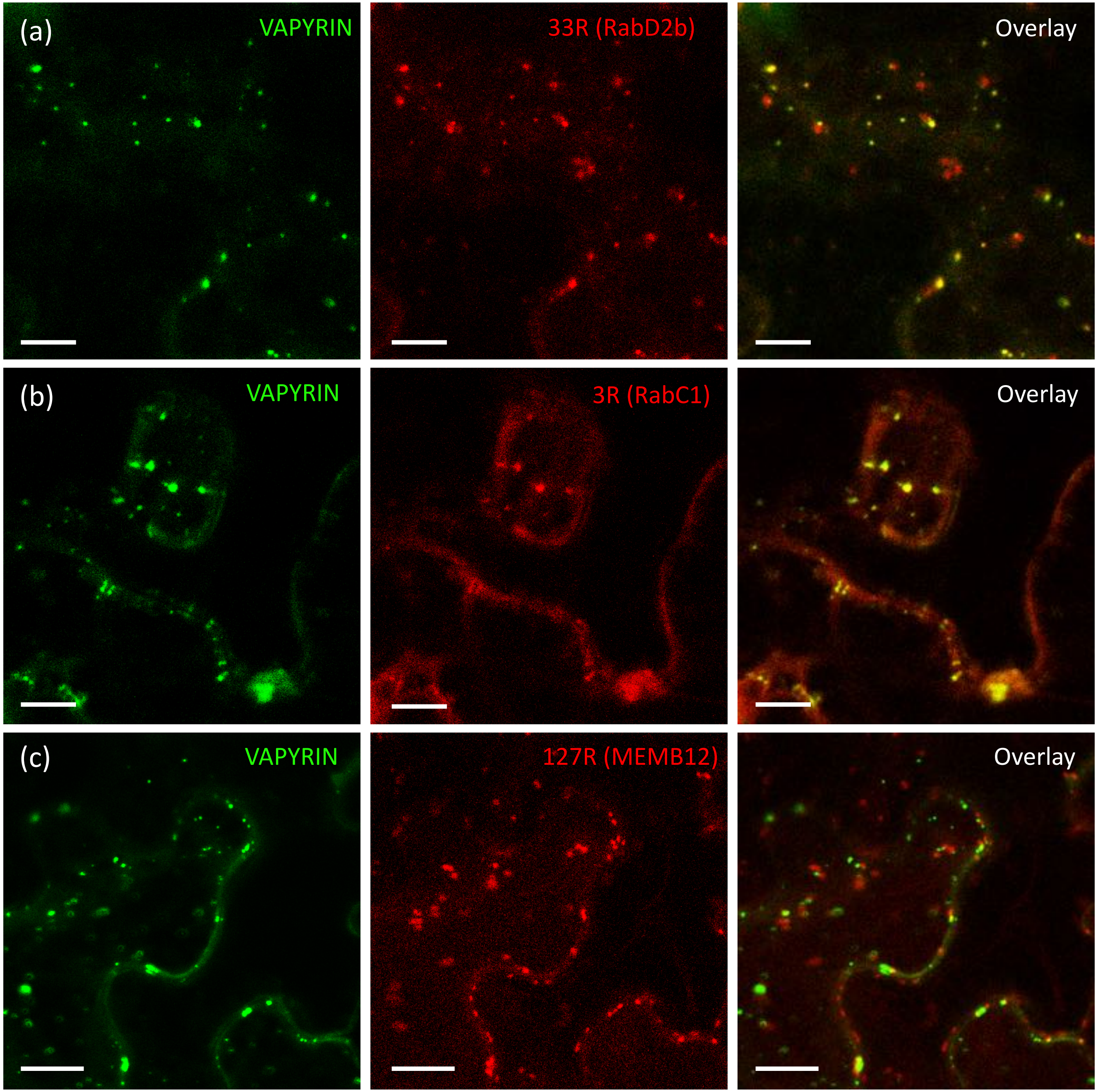

Figure S2 | Co-localization of VAPYRIN with endosomal markers. Co-expression of VAPYRIN-GFP with the endosomal marker mCherry-RabD2b (a), with the trans-Golgi/endosomal marker mCherry-RabC1 (b), and with the Golgi marker mCherry-MEMB12 (c). Size bar: 10µm.

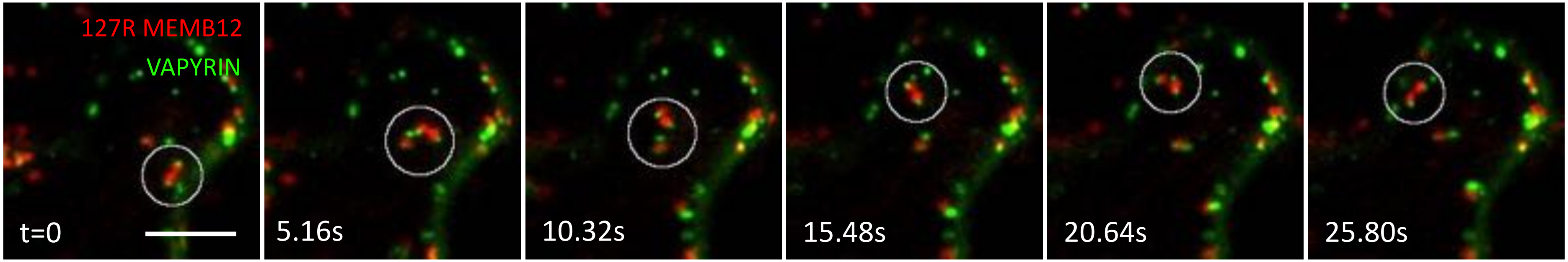

Figure S3 | Association of Vapyrin-bodies with Golgi stacks. A consecutive series of images from a movie reveals the long-term association of Vapyrin-bodies with MEMB12-compartments. Time steps are 5.16 seconds, a white circle highlights a small group of paired compartments. Size bar: 10µm.

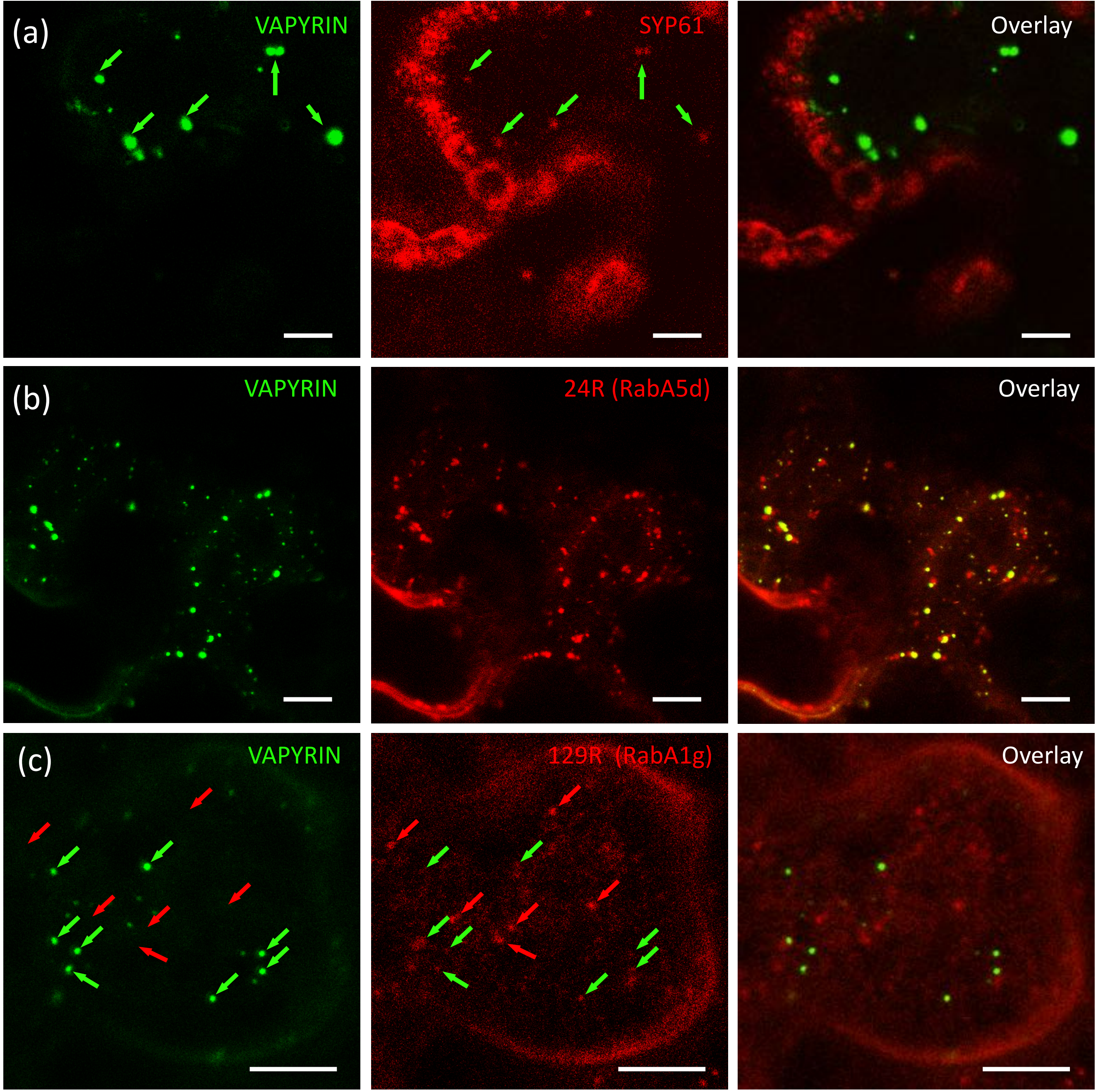

Figure S4 | Co-localization of VAPYRIN with markers for post-Golgi and recycling endosome. Co-expression of VAPYRIN-GFP with the post-Golgi marker mCherry-SYP61 (a), with the trans-Golgi/endosomal marker mCherry-RabA5d (b), and with the endosomal/recycling endosome marker mCherry-RabA1g (c). Size bar: 10µm.

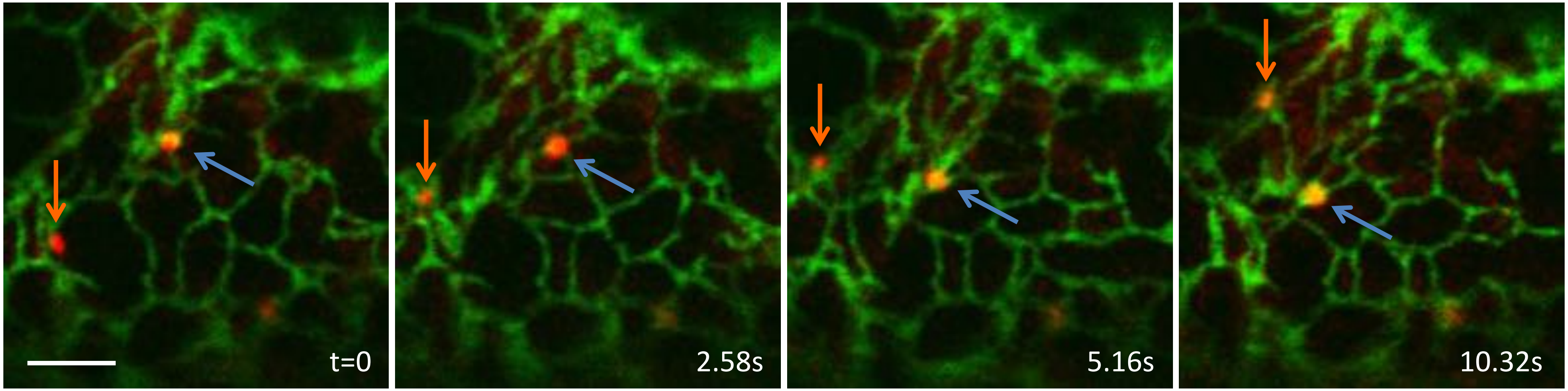

Figure S5 | Association of Vapyrin-bodies with the ER system. A series of images from a movie reveals the association of Vapyrin-bodies (arrows; marked with VAPYRIN-RFP) with the endoplasmic reticulum (marked with GFP-HDEL), and their movement along ER strands (orange arrow). Time steps are indicated. Size bar: 5µm.

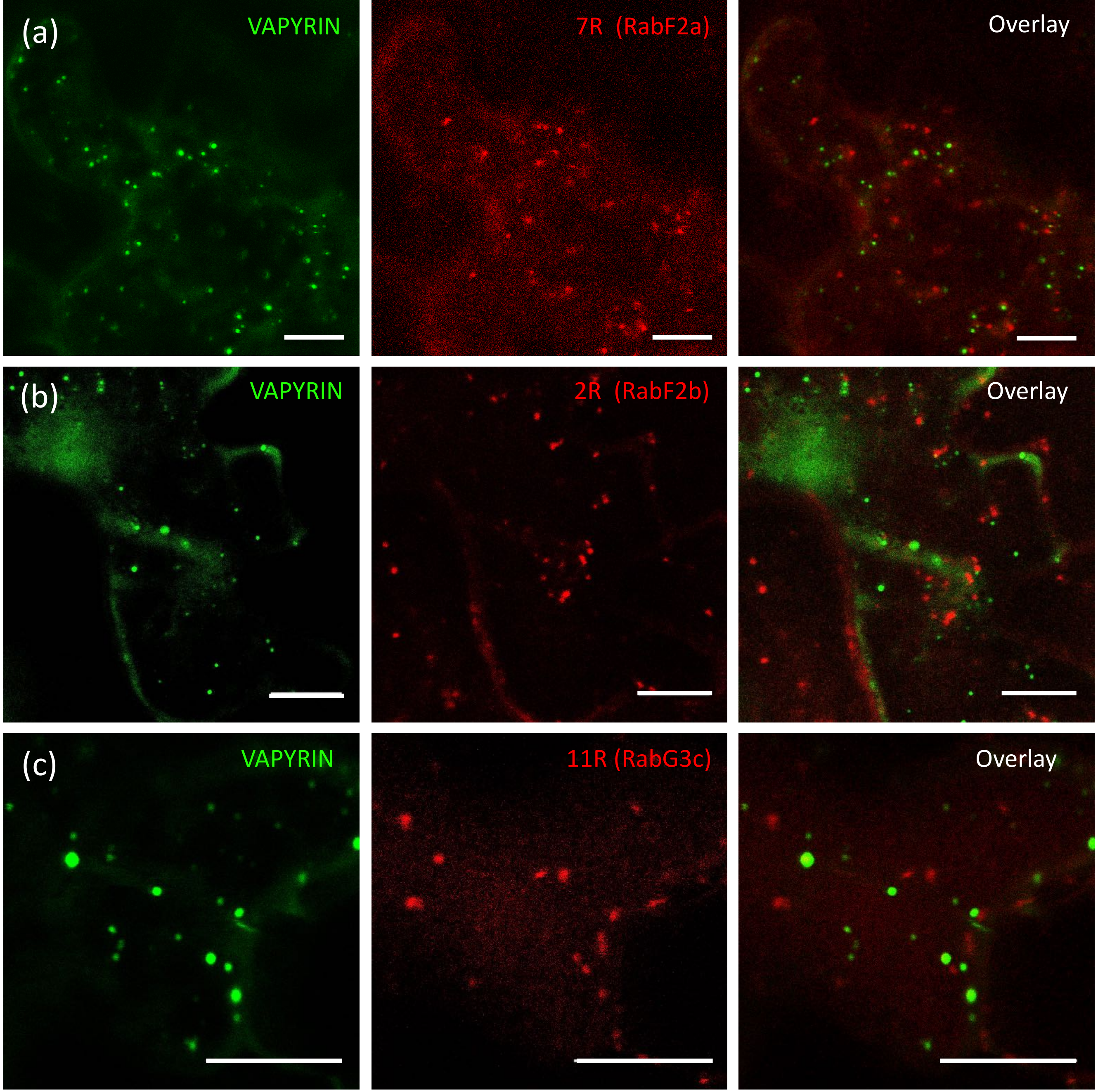

Figure S6 | Co-expression of VAPYRIN with pre-vacuolar markers. Co-expression of VAPYRIN-GFP with the late endosomal/prevacuolar marker mCherry-RabF2a (a), with the late endosomal/pre-vacuolar marker mCherry-RabF2b (b), and with the late endosomal/vacuolar marker mCherry-RabG3c (c). Size bars: 10µm.

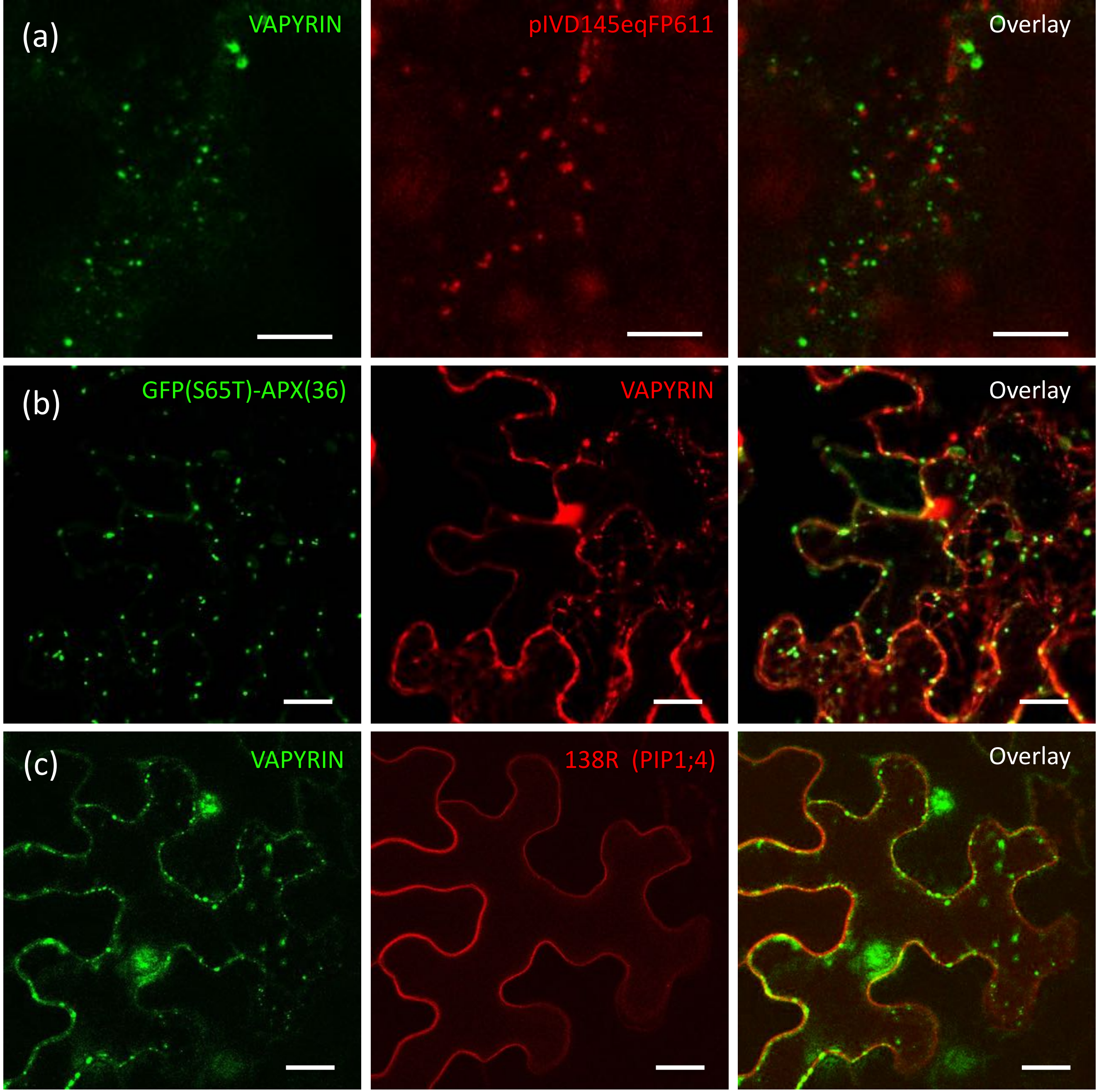

Figure S7 | Co-expression of VAPYRIN with non-endosomal markers. Co-expression of VAPYRIN-GFP with the mitochondrial marker pIVD145seqFP611 (a), with the peroxisomal marker GFP(S65T)-APX(36) (b), and with the plasma membrane marker RFP-PIP1;4 (c). Size bar: 10µm.

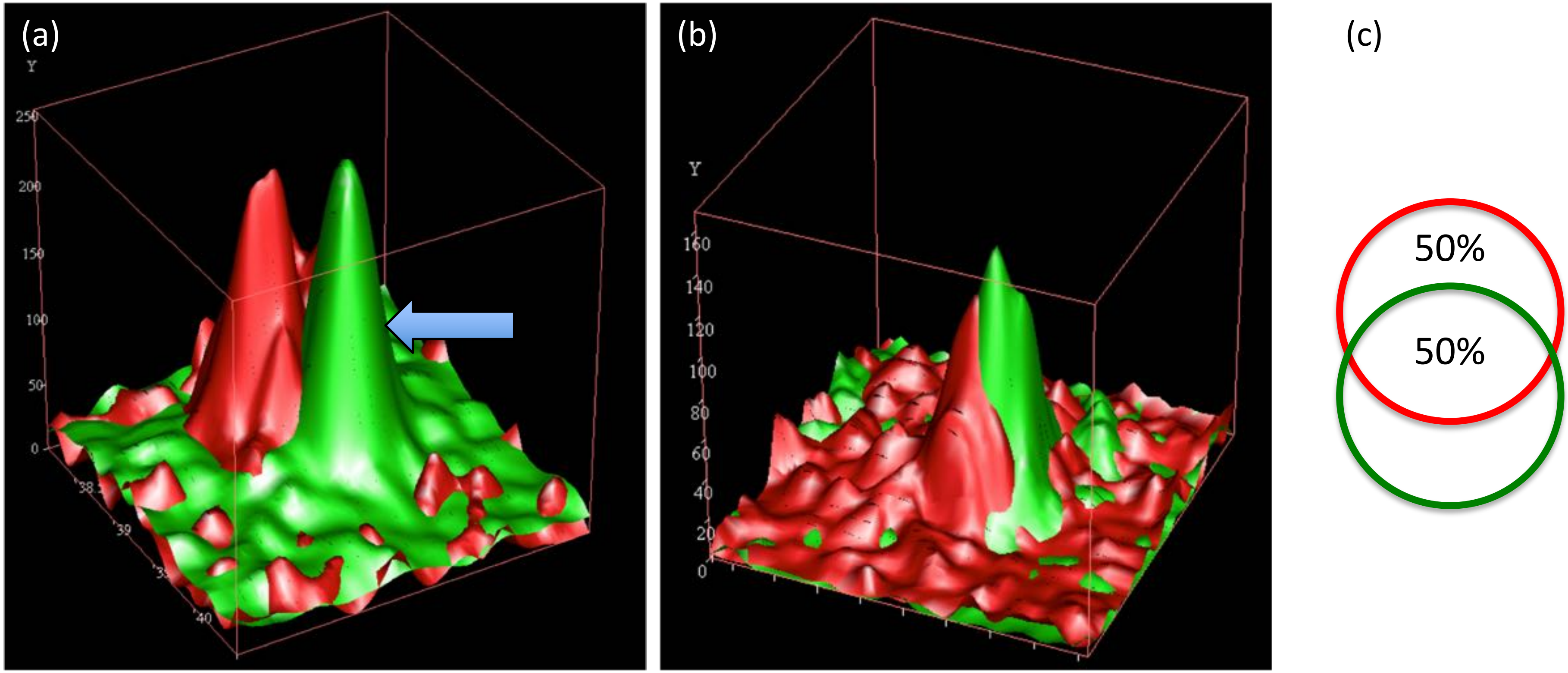

Figure S8 | Concept of quantification of colocalization. (a) Intensity plot of an image with two adjacent nearly circular objects in the green and red channel, respectively. At the half intensity level (blue arrow) the objects are hardly touching. (b) Intensity plot as in (a) with two objects that are virtually overlapping. (c) Definition of the threshold level for co-localization. Objects were considered to co-localize if their cross-section at half-height intensity overlapped by at least 50%.
