# Supplementary material for: VAPYRIN Marks an Endosomal Trafficking Compartment Involved in Arbuscular Mycorrhizal Symbiosis
Source: Front Plant Sci. 2019 Jun 4;10:666. doi: 10.3389/fpls.2019.00666 (PMC6558636; doi:10.3389/fpls.2019.00666)
Supplement: Figure S9 — Preparation of microsomal membranes and attempted solubilization of VAPYRIN-GFP. (a) Western blot with monoclonal anti-GFP antibody using N. benthamiana leaves expressing VAPYRIN-GFP. After removal of crude cellular debris, the supernatant (input) was ultracentrifuged to yield microsomal membranes (MEM) and soluble supernatant (SP). The MEM fraction was then treated with 1% NP-40 in order to solubilize VAPYRIN-GFP, but it remained entirely associated with the MEM fraction. (b) Same procedure as in (a), but with leaves expressing ER-localized GFP-HDEL. (c) Same procedure as in (a), but with leaves expressing free cytoplasmic GFP. [file Data_Sheet_2.pdf]

Figure S9. Preparation of microsomal membranes and attempted solubilization of VAPYRIN-GFP.

(a) Western blot with monoclonal anti-GFP antibody using *N. benthamiana* leaves expressing VAPYRIN-GFP. After removal of crude cellular debris, the supernatant (input) was ultracentrifuged to yield microsomal membranes (MEM) and soluble supernatant (SP). the MEM fraction was than treated with 1% NP-40 in order to solubilize VAPYRIN-GFP, but it remained entirely associated with the MEM fraction.

(b) Same procedure as in (a), but with leaves expressing ER-localized GFP-HDEL.

(c) Same procedure as in (a), but with leaves expressing free cytoplasmic GFP.

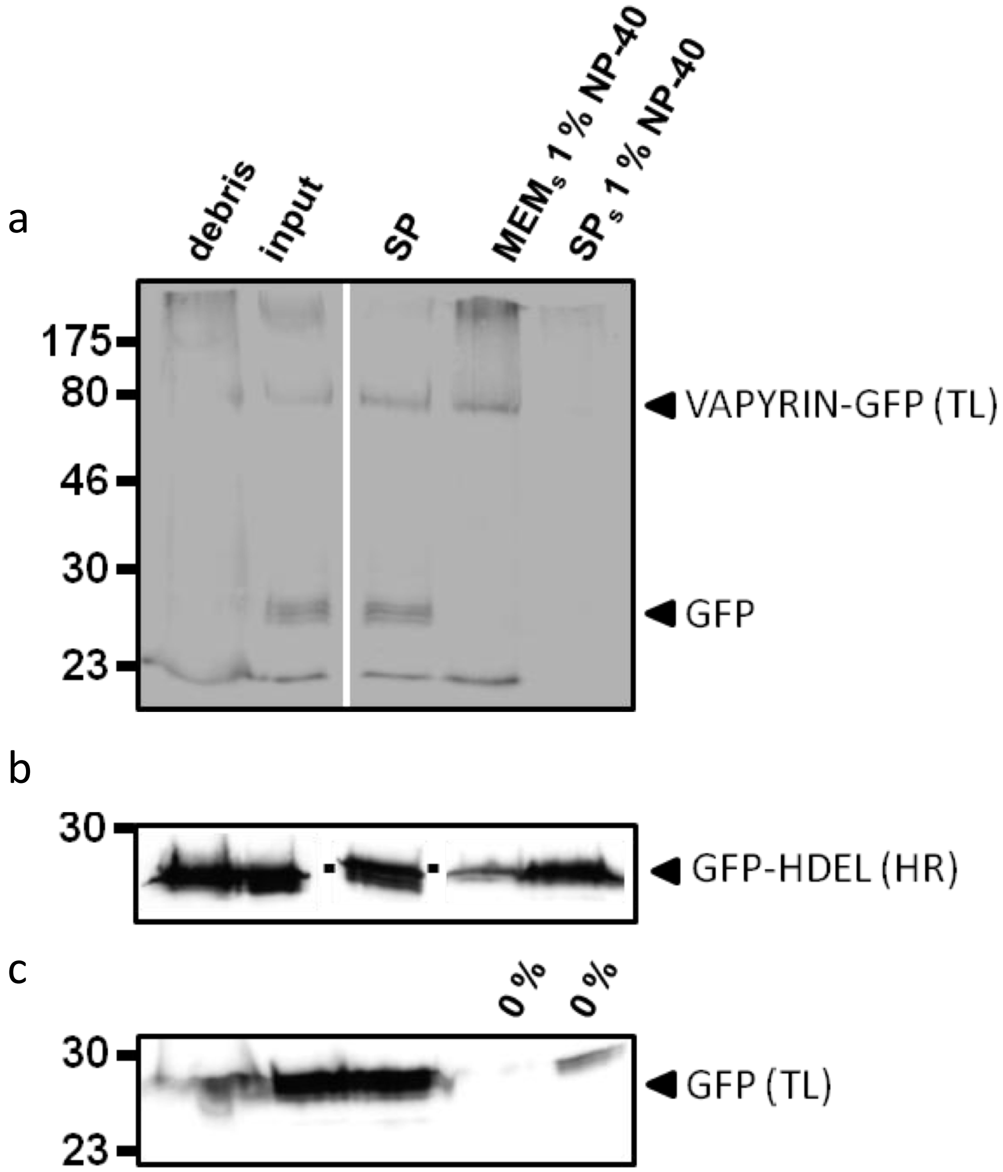

	Small scale fractionation			Large scale fractionation
	Solubilization Buffer A pH 7.5	Solubilization Buffer B pH 7.9	Solubilization Buffer C pH 7.5	Solubilization Buffer C pH 7.5
**NP-40**	1; 3; 5 %	5 %	5 %	5 % (1 % *Ph*VAPYRIN-GFP)
**Triton X-100**	1.5; 3 %		0.5 %, 1%	
**Octyl glucoside**	1 %		2; 5%	
**SDS**	2 %			
**other**	pH 6.8; 9.5			

Figure S10. Experimental conditions in tissue fractionation for the extraction and solubilization of VAPYRIN-GFP and VAP-GFP. Transgenic hairy roots of *P. hybrida* were used to extract membrane pellets with a large scale and a small scale protocol (see Materials and Methods). Subsequently, the membranes were resuspended in different buffers containing various concentrations of different detergents as indicated.

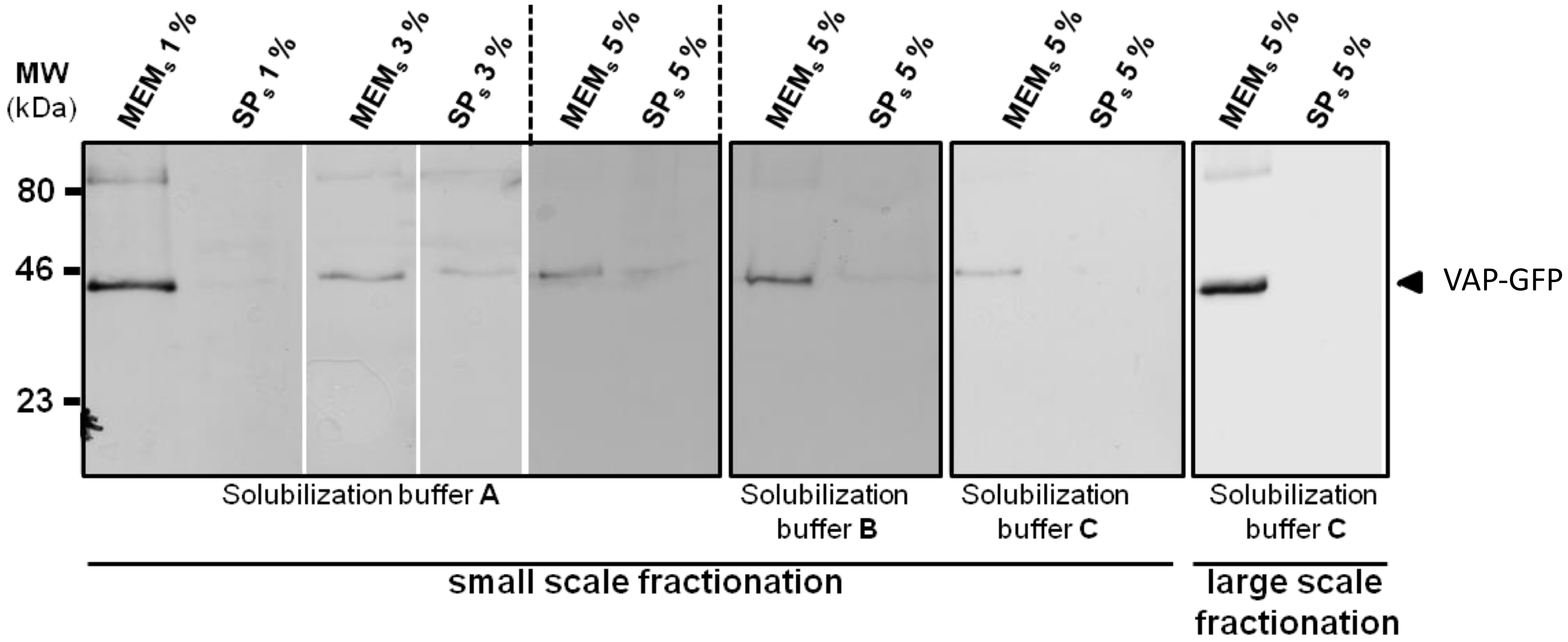

Figure S11. Attempted solubilization of VAP-GFP with NP-40.

Microsomal membrane fractions prepared from hairy roots expressing VAP-GFP were resuspended in diffent buffers with various concentrations of NP-40 as indicated. After incubation for 1h at 4°C, the membrane pellets (MEM) were recovered by centrifugation and subjected to Western blot analysis next to the respective soluble fraction (SP; supernatant after detergent treatment). In all cases, most of the VAP-GFP remained associated with the membranes.

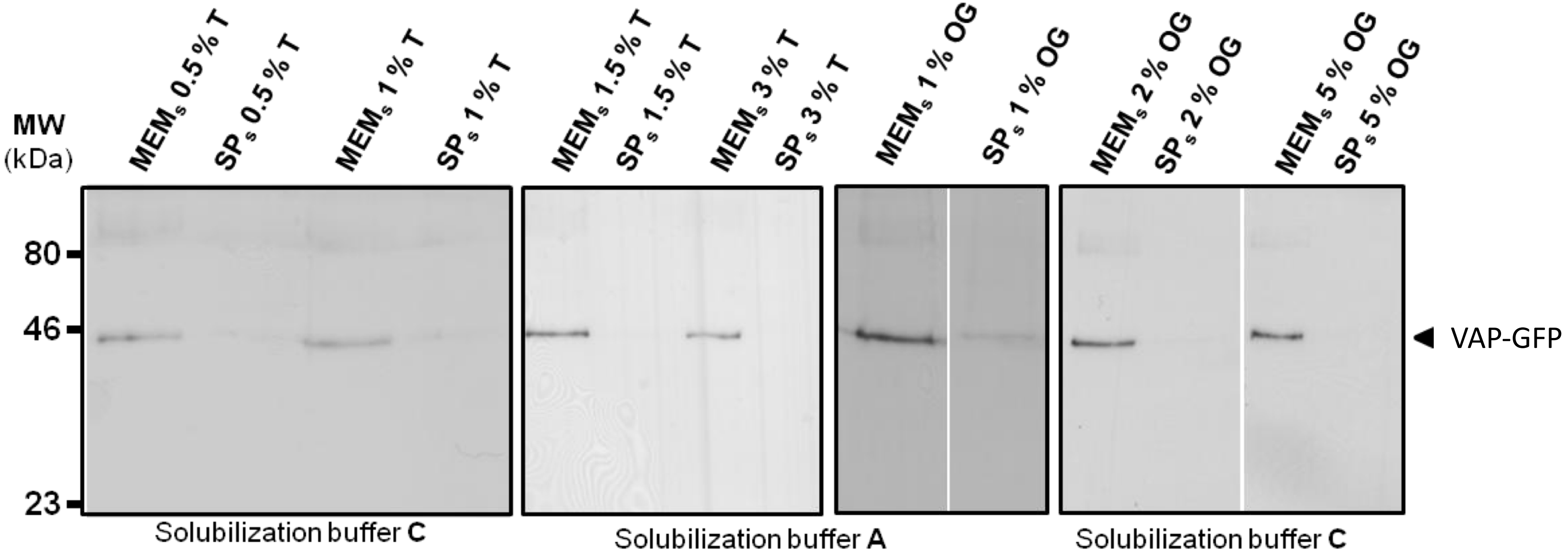

Figure S12. Attempted solubilization of VAP-GFP with Triton-X-100 and octylglucoside.
Microsomal membrane fractions prepared from hairy roots expressing VAP-GFP were resuspended in diffent buffers with various concentrations of Triton-X-100 (T), or octylglucoside (OG) as indicated. After incubation for 1h at 4° C, the membrane pellets (MEM) were recovered by centrifugation and subjected to Western blot analysis next to the respective soluble fraction (SP; supernatant after detergent treatment). In all cases, most of the VAP-GFP remained associated with the membranes.

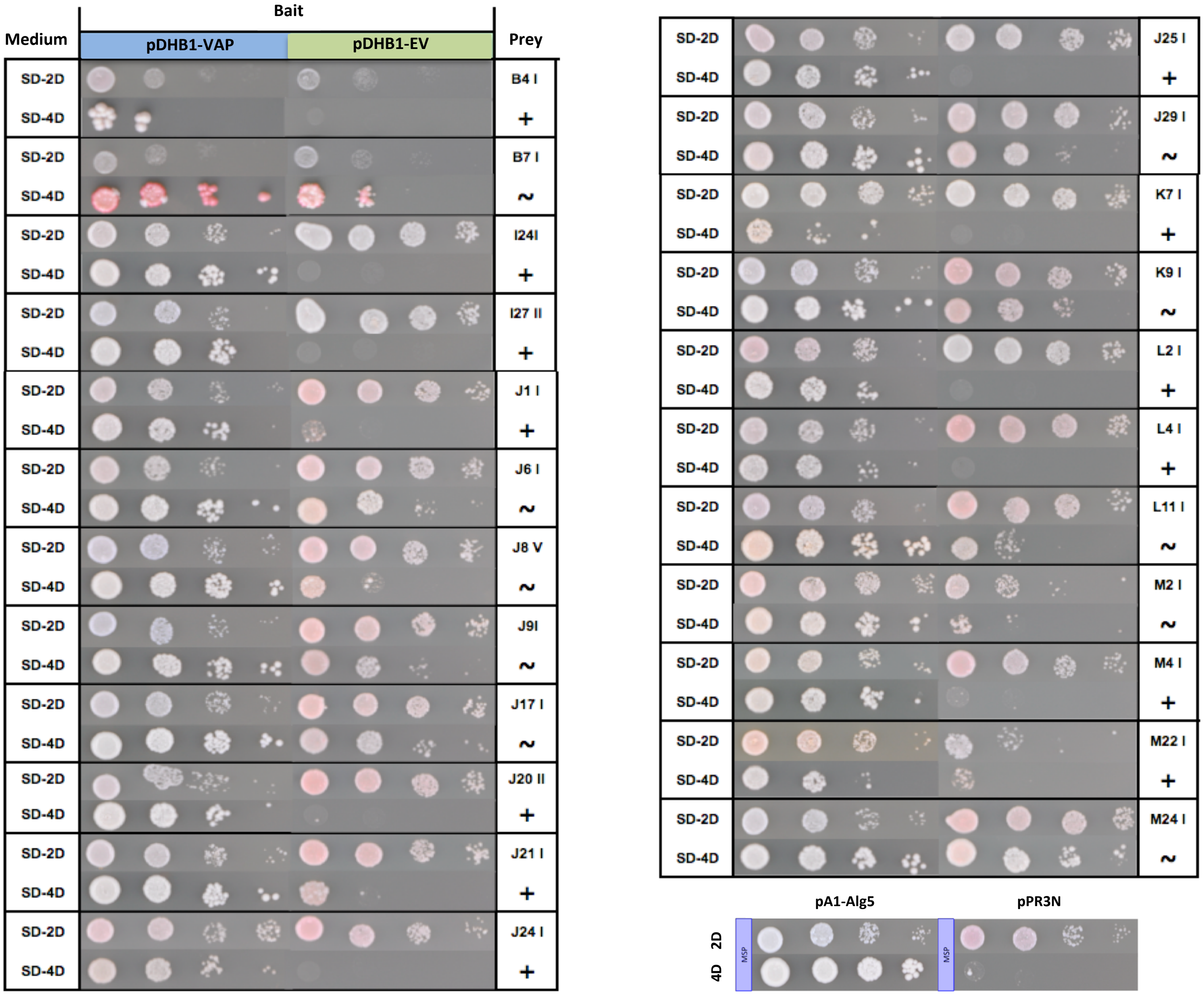

Figure S13. Drop test with interactor candidates from split-ubiquitin screen. Interaction between the bait and the respective candidates is revealed by the white color and the better growth on selective medium (SD-4D) with the bait (pDHB1-VAP), relative to the empty vector control (pDHB1-EV). Growth on non-selective medium (SD-2D) is independent of an interaction and just reflects the presence of the bait and prey vectors. Highly specific interactors are signified by a + sign, moderately specific interactors are indicated with a ~ sign. A positive control is provided by pA1-Alg5 vs. pPR3N.

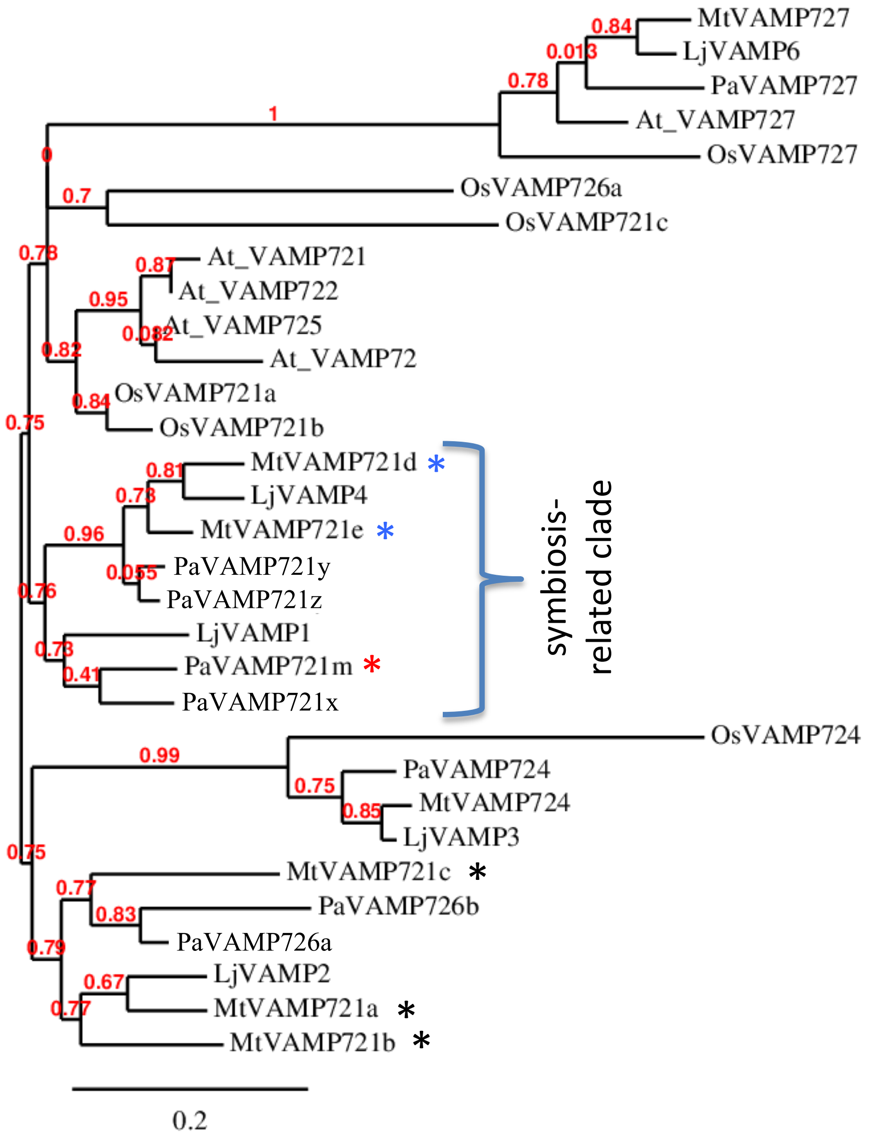

Figure S14. Phylogenetic analysis of petunia VAMP721.

The petunia VAMP721m (red asterisk) in this study was compared with all related VAMPs from *Petunia axillaris* (Pa), *Medicago truncatula* (Mt), *Lotus japonicus* (Lj), *Oryza sativa* (Os), and *Arabidopsis thaliana* (At). Symbiosis-related (blue asterisks), and non-symbiotic (black asterisks) VAMP721 members from *M. truncatula* are indicated.

Table S1. List of subcellular fluorescent makers employed in this study

Protein Name	Wave No.	Assignment localization	Interaction with Vapyrin bodies	Source/Reference
**Rab C1**	3R	Post-Golgi/endosomal	Colocalization	Geldner *et al.*, 2009
**Rab A5d**	24R	Endosomal/recycling endosome	Colocalization and association	Geldner *et al.*, 2009
**Rab D2b**	33R	Golgi/endosomal	Colocalization and association	Geldner *et al.*, 2009
**Rab A1g**	129R	Endosomal/recycling endosome	No interaction	Geldner *et al.*, 2009
**Rab G3c**	11R	Late endosome/vacuole	No interaction	Geldner *et al.*, 2009
**RabF2b (ARA7)**	2R	Late endosome/pre-vacuolar compartment	No interaction	Geldner *et al.*, 2009
**RabF2a (Rha1)**	7R	Late endosome/pre-vacuolar compartment	No interaction	Geldner *et al.*, 2009
**gamma-TIP**		tonoplast	No interaction	Hunter et al., 2007
**delta-TIP**		tonoplast	No interaction	Hunter et al., 2007
**MEMB12**	127R	Golgi	Association	Geldner *et al.*, 2009
**VAMP721**		Early endosome	Colocalization	This study
**SYP61**		Trans-Golgi network	Colocalization	(Drakakaki et al., 2012; Hachez et al., 2014)
**AtG8a**		Autophagosomes	No interaction	(Zhuang et al., 2017)
**PIP1;4**	138R	Plasma membrane	No interaction	Geldner *et al.*, 2009
**pIVD145-eqFP611**		Mitochondria	No interaction	(Forner and Binder, 2007)
**GFP(S65T)-APX(36)**		Peroxisomes	No interaction	(Forner and Binder, 2007)
**GFP-HDEL**		ER	Association	VIB, Belgium

Table S2. Statistical analysis of quantitative co-localization analysis.

	ANK-GFP	VAP-GFP	VAPYRIN-RFP	RabD2b	RabC1	RabA5d	SYP61	MEMB12	RabA1g	RabF2a	eqFP611
ANK domain	1.000	1.000	1.000	1.000	1.000	1.000	**0.011**	**0.000**	**0.000**	**0.000**	**0.000**
VAP domain	1.000	1.000	1.000	1.000	1.000	1.000	0.144	**0.000**	**0.000**	**0.000**	**0.000**
VAPYRIN	1.000	1.000	1.000	1.000	1.000	1.000	0.388	**0.001**	**0.000**	**0.001**	**0.000**
RabD2b	1.000	1.000	1.000	1.000	1.000	1.000	0.569	**0.001**	**0.000**	**0.001**	**0.000**
RabC1	1.000	1.000	1.000	1.000	1.000	1.000	0.992	0.098	0.054	**0.089**	**0.019**
RabA5d	1.000	1.000	1.000	1.000	1.000	1.000	1.000	0.468	0.331	0.448	0.206
SYP61	**0.011**	0.144	0.388	0.569	0.992	1.000	1.000	0.953	0.821	0.898	0.412
MEMB12	**0.000**	**0.000**	**0.001**	**0.001**	0.098	0.468	0.953	1.000	1.000	1.000	1.000
RabA1g	**0.000**	**0.000**	**0.000**	**0.000**	0.054	0.331	0.821	1.000	1.000	1.000	1.000
RabF2a	**0.000**	**0.000**	**0.001**	**0.001**	0.089	0.448	0.898	1.000	1.000	1.000	1.000
eqFP611	**0.000**	**0.000**	**0.000**	**0.000**	**0.019**	0.206	0.412	1.000	1.000	1.000	1.000

Tukey HSD post-hoc test was performed to test for the degree of co-localization. Based on p-values, Two clear groups emerged:
Group A: ANK-GFP, VAP-GFP, VAPYRIN-RFP, RabD2b, RabC1, and RabA5d
Group B: MEMB12, RabA1g, RabF2a, and eqFP611
SYP61 takes an intermediate position.

Table S3. List of interactor candidates identified by the split-ubiquitin screen

ID	Assigned function	protein length	% of protein covered	Signal peptide	Predicted localisation	TM domain
**B4 I**	Nitrilase	281	98%	–	mitochondrial matrix	–
**B7 I**	TA20, anther-related	73	73%	pos. 28	vacuole	–
**I24 I**	Unknown (NAM-like?)	166	17%	?	ER membrane	1
**I27II**	PIP aquaporin	283	98%	–	plasma membrane	6
**J1I**	Copper-binding-like domain	108	54%	–	[illegible] nembrane	1
**J6I**	LAT52	166	94%	pos. 20	extracellular	–
**J8V**	Microsomal signal peptidase	189	98%	–	plasma membrane	2
**J9I**	PIP aquaporin	283	84%	–	plasma membrane	5
**J17 I**	VAMP (R-SNARE)	103	46%	?	ER membrane	1
**J20 II**	Vacuolar proton ATPase	164	100%	uncleavable	vacuolar membrane	4
**J21 I**	Microsomal signal peptidase	189	98%	–	plasma membrane	2
**J24 I**	Bacterial sequence	176	22%	?	–	1
**J25 I**	Hexa-ubiquitin	376	87%	–	cytoplasm	–
**J29 I**	Unknown	291	100%	pos 29	Extracellular	–
**K7 I**	GLP, rhicadhesin receptor	222	99%	pos. 22	cell wall	–
**K9 I**	Lesion-inducing protein	157	100%	pos. 22	plasma membrane	2 or 3
**L2 I**	TIP aquaporin	246	99%	pos. 39	plasma membrane	6
**L4 I**	Unknown (RING-domain)	218	92%	–	ER membrane	3
**L11 I**	LAT52	166	94%	pos. 20	extracellular	–
**M2 I**	Peptidase inhibitor	93	100%	–	cytoplasm	–
**M4 I**	PIP aquaporin	287	100%	–	plasma membrane	6
**M22 I**	Sucrase /ferredoxin-like	287	77%	–	cytoplasm	–
**M24 I**	VAMP-like SEC22 (R-SNARE)	62	27%	?	ER membrane	1

Table S4. RNAseq data for 12 VAMP genes of *P. hybrida* in mycorrhizal roots vs. control roots

		RPKM values									
Feature ID		M1	M2	M3	**M ave**	c1	c2	c3	**c ave**	p-value*	**M/c***
Peaxi162Scf00107g01117.1	VAMP721m	384	397	449	**410**	545	430	509	**495**	0.12	**0.83**
Peaxi162Scf00517g00424.1	VAMP721x	792	800	921	**838**	741	702	855	**766**	0.06	**1.09**
Peaxi162Scf00149g01112.1	VAMP721y	1349	1465	1798	**1537**	1957	1865	2099	**1974**	0.00	**0.78**
Peaxi162Scf00367g00010.1	VAMP721z	895	920	1028	**948**	1113	1076	1161	**1117**	0.00	**0.85**
Peaxi162Scf00047g01029.1	VAMP726a	0	0	0	**0**	0	0	0	**0**	1.00	**n.d.**
Peaxi162Scf00817g00123.1	VAMP726b	0	0	1	**0**	0	0	0	**0**	0.58	**n.d.**
Peaxi162Scf00125g00227.1	VAMP727	627	622	741	**663**	613	660	783	**685**	1.00	**0.97**
Peaxi162Scf00534g00007.1	VAMP724	115	88	88	**97**	113	95	78	**95**	1.00	**1.02**
Peaxi162Scf00643g00112.1	VAMP713	532	551	648	**577**	623	737	886	**749**	0.11	**0.77**
Peaxi162Scf00136g00229.1	VAMP711	309	323	379	**337**	336	399	476	**404**	0.38	**0.83**
Peaxi162Scf00091g00616.1	VAMP714a	276	323	366	**322**	315	276	359	**317**	0.82	**1.02**
Peaxi162Scf00091g00617.1	VAMP714b	178	215	230	**208**	210	181	226	**206**	0.85	**1.01**

*Baggerley's test: M vs. c original values - FDR p-value correction

**Induction ratio mycorrhizal/control
