# Supplementary material for: VAPYRIN Marks an Endosomal Trafficking Compartment Involved in Arbuscular Mycorrhizal Symbiosis
Source: Front Plant Sci. 2019 Jun 4;10:666. doi: 10.3389/fpls.2019.00666 (PMC6558636; doi:10.3389/fpls.2019.00666)
Supplement: File S1 — Protein sequences used for phylogenetic analysis of VAMP proteins. [file Data_Sheet_3.PDF]

>PaVAMP721m
MGQQSLIYSFVARGTVILAEYTEFTGNFTSIASQCLQKLPASNNKFTYNCDGHTFNYLVDDGYTYCVVA
VESVGRQIPIAFLERTKEEFTKKYGGGKAATAVANSLNREFGPKLKEQMQYCVDHPEEISKLSKVKAQV
SEVKGVMMENIEKVLDRGEKIELLVDKTENLRSQAQDFRTQGTTMRRKMWLQNMKIKLIVLGIIIALIL
IIVLSACGGFKCH

>PaVAMP721x
MGQQSLIYSFVARGTVIVAEFTEFTGNFTSIASQCLQKLPASNNKFTYNCDGHTFNYLVDNGYTYCVVA
VESVGRQVPIAYLERIKDDFTKKYGGGKAATAVANSLNKEFGPKLKEQMQYCVDHPEEISKLAQVKAQV
SEVKGVMMENIEKVEQYPLFYCLKGADVLTWETTKALKFKKLLWGLLIRIVALQVLDRGEKIELLVDKT
ENLRSQAQDFRTQGTKVRRKMWLKNMKIKLIVLAIIIALILVIVLSICHGFKCH

>PaVAMP721y
MGQQKALIYSFVGRGNVILAEHTDFSGNFNSIAYQCLQKLPASNNKFTYNCDGHTFNYLVDNGFTYCVV
AEESVGRQIPIAFLERVKDDFVSKYGGGKAATAPPNSLNKEFGPKLKEHMQYCADHPEEISKLAKVKAQ
VSEVKGVMMENIEKVLDRGEKIELLVDKTENLHHQAQDFRNTGTQIRRKMWLQNMKIKLIVLGILIALI
LIIVLSACKGFNCGK

>PaVAMP721z
MGQQKVLIYAFVARGNVILAEFTDFSGNFNSIAFQCLQKLPASNNKFTYNCDGHTFNYLVDNGFTYCVV
AEESVGRQVPIAFLERVKDDFVSKYGGGKAATAAPNSLNKEFGPKLKEHMHYCADHPEEISKLAKVKAQ
VSEVKGVMMENIEKVLDRGEKIELLVDKTENLHHQAQDFRNTGTQIRRKMWLQNMKVKLIVLGILIALI
LIIVLSVCHGFNCGK

>PaVAMP726a
MGQQTLIYSFVARGTVILAEYTEFTGNFNTIASQCLQKLPASNNRFTYNCDGHTFNFLAENGFTYCVVA
TESAGRQIPIAFLERIKDDFTKRYGGGKAATATAKSLNKEFGPKLKEHMKYCVEHPEEISKLAKVKAQV
SEVKGVMMQNIEKAQDFRQQGTKIRRKLWYENMKIKLIVLGIIIALILIIIILSCPGFNCF

>PaVAMP726b
MTSKTLIYSFVARGTIILAEYSEFTGNFNSIASQCLQKLPASSNKFSYNCDGHTFNFLSDNGFSREIPL
AFLDRVKDDFVKRYGGGKAATASAKSLNKEFGSKLKEHMKYCVEHPEEISKLSKVKAQVSEVKGVLDRG
EKIELLVDKTENLRSQAQDFRQQGTKIRRKMWYENMKIKL

>PaVAMP727
MNPKGLIYSFVARGTVVLAEHTPYSGNFSTIAVQCLQKLPSNSSKYTYSCDGHTFNFLLDSGFVFLVVA
DESTGRGVPFVFLERVKDDFKKRYSSSIRNDDDPHPLADEEDDDLFGDRFSIAYNLDREFGPRLKEHME
YCMNHPDEMSKLSKLKAQITEVKGIMMDNIEKVLDRGEKIELLVDKTENLQFQADSFQRQGRQLRRKMW
FQNLQMKLMVGGAILVFIIIVWLFACGGFKC

>PaVAMP724
MGQESFIYSFVARGTMVLAEYTEFTGNFPAIAAQCLQKLPSSNNKFTYNCDHHTFNFLVQDGYAFCVVA
KESAGKQISIAFLERVRADFTKRYGGGKADTAVAKSLNKEFGPIMKEHMQYIIDHADEIEKLLKVKAQV
SEVKSIMLENIDKAIERGENLTILNDKAEDLRDSVGPRLQEKGDSNTEEDVVSEYENKSKYLRFRGDDY

>At_VAMP727
MSQKGLIYSFVAKGTVVLAEHTPYSGNFSTIAVQCLQKLPTNSSKYTYSCDGHTFNFLVDNGFVFLVVA
DESTGRSVPFVFLERVKEDFKKRYEASIKNDERHPLADEDEDDDLFGDRFSVAYNLDREFGPILKEHMQ
YCMSHPEEMSKLSKLKAQITEVKGIMMDNIEKVLDRGEKIELLVDKTENLQFQADSFQRQGRQLRRKMW
LQSLQMKLMVAGAVFSFILIVWVVACGGFKCSS

>At_VAMP725
MGQQNLIYSFVARGTVILVEYTEFKGNFTAVAAQCLQKLPSSNNKFTYNCDGHTFNYLVENGFTYCVVA
VESVGRQIPMAFLERVKEDFNKRYGGGKATTAQANSLNREFGSKLKEHMQYCVDHPDEISKLAKVKAQV
TEVKGVMMENIEKVLDRGEKIELLVDKTENLRSQAQDFRTQGTKIRRKMWFENMKIKLIVLGIIITLIL
IIILSVCGGFKCT

>At_VAMP721
MAQQSLIYSFVARGTVILVEFTDFKGNFTSIAAQCLQKLPSSNNKFTYNCDGHTFNYLVEDGFTYCVVA
VDSAGRQIPMSFLERVKEDFNKRYGGGKAATAQANSLNKEFGSKLKEHMQYCMDHPDEISKLAKVKAQV
SEVKGVMMENIEKVLDRGEKIELLVDKTENLRSQAQDFRTTGTQMRRKMWLQNMKIKLIVLAIIIALIL
IIVLSVCHGFKC

>At_VAMP722
MAQQSLIYSFVARGTVILVEFTDFKGNFTSIAAQCLQKLPSSNNKFTYNCDGHTFNYLVENGFTYCVVA
VDSAGRQIPMAFLERVKEDFNKRYGGGKAATAQANSLNKEFGSKLKEHMQYCMDHPDEISKLAKVKAQV
SEVKGVMMENIEKVLDRGEKIELLVDKTENLRSQAQDFRTQGTQMRRKMWFQNMKIKLIVLAIIIALIL
IIILSICGGFNCGK

>At_VAMP72
MGQQSLIYSFVARGTVILAEYTEFKGNFTSVAAQCLQKLPSSNNKFTYNCDGHTFNYLADNGFTYCVVV
IESAGRQIPMAFLERVKEDFNKRYGGGKASTAKANSLNKEFGSKLKEHMQYCADHPEEISKLSKVKAQV
TEVKGVMMENIEKVLDRGEKIELLVDKTENLRSQAQDFRTQGTKMKRKLWFENMKIKLIVFGIIVALIL
IIILSVCHGFKCT

>MtVAMP721a
MGQQSLIYSFVARGTVILAEYSDFTGNFTTIALQCLQKLPASNNRFTYNCDGHTFSFLVDNGFTYCVVA
VESVGRQIPIAFLERIKDDFNKRYGGGRATTATAKSLNKEFGPKLKEQMQYCVEHPEEVSKLAKVKAQV
SEVKGVMMENIDKVIDRGEKIEVLVDKTENLRSQAQDFRQQGTQLRRKMWYQNMKIKLIVLAIIIALIL
IIVLSVCHGFSC

>MtVAMP721b
MGQQSLIYSFVARGTVILAEYTEFTGNFTAVAAQCLQKLPSSNNKFNYNCDDHTFNYLVDSGFTYCVVA
VESAGRQIPIAFLERIKEDFSKKYAGGKAENAAAKSLNKEFGSKLKEQMQYCVEHPEEISKLSKVQAQV
SEVKGVMMENIEKVLDRGEKIELLVDKTENLRSQAQDFRQHGTKLRRKMWFQNMKIKLIVLGIIIALIL
IIVLSICGGFNCSK

>MtVAMP721c
MVQQSLIYSFVARGMVILAEHTNFTGNFVEIALQCLQRLPATNTKFTYNTDGHTFNYLAHDGFTYCVVG
VESFDRHIAMAFLDRIKEDFTKRYGGGKAATATSKSLNKEFGPKLKEHMQYCVEHPEEVSKLAKVKAQV
SQVQDVMLENIDQVLNRQVKIDVLMDKTDNLRDQAQVFRREGGQLRRKMWFQNMKIKLIVLAIIIVIIL
IIVLLVT

>MtVAMP721d
MANNQNQKQLIYAFVSRGTVILAEFTEFSGNFNSIAFQCLQKLPSTNNKFTYNCDNHTFNYLIDNGYTY
CVVADETTGRQVPMAFLERVKDDFVSKYGGEKASTAPPNSLNKEFGPKLKEHMQYCVDHPDEISKLAKV
KAQVSEVKGVMMENIEKVLDRGEKIELLVDKTDNLHHQAQDFRSSGTSIRRKMWLQNMKVKLIVLGILI
ALILIIVLSVTRG

>MtVAMP721e
MGQNQKSLIYAFVSRGSVILSEYTEFSGNFNSIAFQCLQKLPASNNKFTYNCDGHTFNYLVDNGYTYCV
VADETVGRQVPVAFLERVKDDFVAKYGGGKASTAAPNSLNKEFGPKLKEHMQYCVDHPEEVSKLAKVKA
QVSEVKGVMMENIEKVLDRGEKIELLVDKTENLHHQAQDFRNSGTKIRRKMWLQNMKIKLIVLAILIAL
ILIIVLPIVLKNK

>MtVAMP724
MSQESFIYSFVARGTMVLAEYTEFTGNFPAIAAQCLQKLPSSNNKFTYSCDHHTFNFLVEDGYAYCVVA
KESVSKQISIAFLERVKADFKKRYGGGKADTAIAKSLNKEFGPVMKEHMKYIIDHAEEIEKLLKVKAQV
SEVKSIMLENIDKAIDRGENLSVLSDKTETLRAQAQDFRKQGTQVRRKMWYQNMKIKLVVLGILLFLVL
VIWLSICGGFNCSN

>MtVAMP727
MSQRGLIYSFVAKGTVVLAEHTQYTGNFSTIAVQCLNKLPSNSTKYTYSCDGHTFNFLLDNGFVFLVVA
DESIGRSVPFVFLERVKDDFNQRYGASIKIASDHPLADDDEDDDLFEDRFSIAYNLDREFGPSLKGHMQ
YCLTHPEEMSKLSKLKAQITEVKGIMMDNIEKVLIVGRRLNFWWIKRKTCSSRLTASRGRAGS

>OsVAMP721a
MGQQSLIYAFVARGTVVLAEYTEFTGNFTTIAAQCLQKLPASNNKFTYNCDGHTFNYLVEDGFTYCVVA
VESVGRQIPIAFLDRVKEDFTKRYGGGKAATAAANSLNREFGSKLKEHMQYCVDHPEEISKLAKVKAQV
SEVKGVMMENIEKVLDRGEKIELLVDKTENLRSQAQDFRQQGTKVRRKMWLQNMKIKLIVLGIIIALIL
IIILSVCHGFKCK

>OsVAMP721b
MGQQSLIYAFVARGTVILAEYTEFTGNFTTIASQCLMKLPASNNKFTYNCDGHTFNYLVEDGFTYCVVA
VESVGRQIPIAFLDRVKDDFTKRYAGGKAATAAANSLNRDFGSKLKEHMQYCVDHPEEISKLAKVKAQV
SEVKGVMMENIEKVLDRGEKIELLVDKTENLRSQAQDFRQAGTQVRRKMWLQNMKIKLIVLGIIIALIL
IIILSVCHGFKCK

>OsVAMP726a
MAPQKRTTLVYSFVARGAVVLADHAEVSGNFASVAAQCLQKLPSTNNRHSYNCDGHTFNYHVHDGFTYC
VVATESAGRQLPVGFIERVKEDFSKKYSGGKAKNATANSLKREYGPKLKEHMKYCDAHPEEIDKLAKVK
AQVTEVKGVMMQNIEKVLDRGEKIELLVDKTEDLRSQAQDFRKAGTKIRRKMWWENMKMKLIVFGIVVA
LILVIILTVCRDLNCW

>OsVAMP721c
MAESKLIYAMVARGTVVLAEHTAYAGNFRDIAAQCLQKLPAGDNRLTYTCDAHTFNFLIHQGYAYCVVA
TESSGRQIPLALLDMIKEDFNKRYAGGKAATAAANSLSRDFGPRLGEQMKYCMDHPEEVSKLAKVKAQV
SEVKGIMMENIDKAIDRGQQIDVLVSRTEQLHDQAADFRQQGTRVRRKMWYQNMKIKLIVLGIIIALIL
IIILSVCHGFKC

>OsVAMP727
MNGNKQSLIYSFVAKGSVVLAEHTAFSGNFSTIAVQCLQKLPPNTSKSTYSCDGHTFNFLVDRGFVFLV
VADEAVGRSVPFVFLDRVKEDFMQRYGSSIDEEGQHPLADDADDDDFLLEDRFSIAYNLDREFGPRLKD
HMLYCINHPEEISKLSKVKAHLTEVKGIMMDNIEKILERGEKIELLVGKTETLQSQADSFHRHGRELRR
KMWLQNLRFKLMVGGAVAALILFLWLIICGGFKC

>OsVAMP724
MASPPGKKGEGGGDGGGGKAEWLIYAFVARGTAVLAEYTEFTGNFPALAAQCLQRLPASGGGGSGGGAP
ARFSYACDGHTFNFLLHRGYAYCVVAKESVPKNVSVAFLERLKDDFMKRYGGGKADTALAKSLNKEYGP
VIKQHMQYVLDHSEEIEKTLKVQAQVSEVKNIMLENIEKTLGRGEKLSELQDKTSDLQSQAQEFKKKGV
KIRRKTWLQNMKIKLVVLGILLLLVIIVWVSVCQGFDCTKH

>LjVAMP1
MGQQTLIYSFVARGTMVLAEFTEFSGNFTSIACQCLQKLPSSNNRFTYNCDGHTFNYLVENGFTYCVVA
VESAGRQIPIAFLERVKEEFSKKYGGGKAATASTHSLNKEFGPKLKQQMQYCVDHPEEISKLAKVKAQV
SEVKGVMMENIEKVLDRGEKIEMLVDKTDNLRSQAQDFRTQGTKMKRRCGFTI

>LjVAMP2
MGQQSLIYSFVARGTLILAEYTDFNGNFTSIALQCLQKLPASNKKFTYNCDGHTFSYLVDNGFTYCVVA
VESFGQQIPMAFLERIKEDFTKKYAGGRASTAAAKSLNKEFGPKLKEQMQYCVEHPEEVNKLAKVKAQV
SEVKEVMMENIEKVLDRGVKIDVLEEKTENLRSQAQDFRQQGTQLRRKMWYQNMKIKLIIILAIIIALIL
IIVLSVCNGFNS

>LjVAMP3
MSQESFIYSFVARGTMVLAEYTEFTGNFPAIAAQCLQKLTSSNNKFTYNCDHHTFNFLVEDGYAYCVVA
KESVSKQISIAFLERVKADFKKRYGGGKADTAVAKSLNKEFGPVMKEHMKYIIDHAEEIEKLIKVKAQV

SEVKSIMLENIDKAIDRGENMTILADKTETLRSQAQDFRKQGTQVRRKMWYQNMKIKLVVFGILLFLVL
VIWLSICGGFDCSN

>LjVAMP4
MGPPNQNQNQKPLIYAFVSRGTVILAEFTEFSGNFNSIAFQCLQKLPSTSNKFTYNCDAHTFNFLVDNG
YTYCIVADESVGRQVPMAFLERVKDDFVSKYGDGKAATAPANSLNKEFGPKLKEHMQYCVDHPEEISKL
AKVKAQVSEVKGVMMENIEKVLDRGEKIELLVDKTENLHHQAQDFRTSGTSIRRKMRLQNMKIKLIVLG
ILIALILIIVLSATRGFQK

>LjVAMP6
MLFNVAEAASNSSKYTYSCDGHTFNFLLDNGFVFLVVADESVGRSVPFVFLERVKDDFMQRYGASIENA
SDHPLADDDDDDDLFEDRFSIAYNLDREFGPALKEHMQYCLNHPEEMSKLSKLKAQITEVKGIMMDNIE
KVLDRGEKIELLVDKTESLQFQADSFQRQGRQLRRKMWLQNLQMKLMVGGGILILVIILWVIACGGFKC
