# Supplementary material for: VAPYRIN Marks an Endosomal Trafficking Compartment Involved in Arbuscular Mycorrhizal Symbiosis
Source: Front Plant Sci. 2019 Jun 4;10:666. doi: 10.3389/fpls.2019.00666 (PMC6558636; doi:10.3389/fpls.2019.00666)
Supplement: File S2 — Supplementary Materials and Methods. [file Data_Sheet_4.PDF]

Supplementary Materials and Methods

Stable transformation of *P. hybrida*

Seeds of *Petunia hybrida* W115 were surface sterilized in 70 % ethanol (1 min), followed by 7% bleach with 0.1% Tween 20, and rinsed 5 times with distilled water before plating on MS medium. Leaf fragments (ca. 0.5cm x 0.5cm) of in vitro grown plants were incubated for 15 min in Petri dishes with 25mL of a *A. tumefaciens* suspension (OD600=0.5) supplemented with 200µM acetosyringon. Then the explants were rinsed in distilled water and briefly dried on filter paper. Twenty to thirty explants per plate were put on co-cultivation MS medium for two days at 25°C in the dark. Then eight to ten explants per plate were transferred to selective medium (MS medium with 250µg·l^{-1} cefotaxime / carbenicillin and appropriate antibiotics, NAA 0.1µg·ml^{-1} and BAP 2µg·l^{-1}) in a growth chamber (photoperiod: 16h, temperatures: day at 25°C, night at 21°C). The plant material was transferred to fresh medium every four weeks. Calli of > 1mm were cut and transferred to shooting medium medium (MS with 250µg·l^{-1} cefotaxime/carbenicillin and appropriate antibiotic, BAP 2µg·ml^{-1}). Shoots >5 mm were cut and transferred to rooting medium (MS with 250µg·l^{-1} cefotaxime / carbecillin and appropriate antibiotic, NAA 0.1µg·ml^{-1}). Then roots of the plantlets were washed to remove solid medium and were transferred to soil in trays with lids to keep a high level of humidity for four weeks.

Stable transformation of *A. thaliana*

Arabidopsis thaliana Col-0 was transformed according to the floral dip method (Zhang et al., 2006). Briefly, the plants were grown under long-day conditions and the first bolts were clipped. Two milliliter precultures of transformed *Agrobacterium tumefaciens* strain GV3101::pMP90 (Bechtold et al., 1993) were incubated overnight in YEB medium with the appropriate antibiotics (28°C, 210 rpm). The day after 400 ml YEB medium with the appropriate antibiotics were inoculated with precultures and incubated overnight (28°C, 210 rpm). At an OD600 of 0.5-1 the agrobacterial cultures were centrifuged at 4500g for 10 min at 4°C. The bacterial pellets were resuspended in infiltration medium (10 mM $MgCl_2$, 5% w/v sucrose, pH=5.7) to obtain an OD600 0.8. Then 0.02 % Silvet L-77 was added and the agrobacterial suspension was mixed gently. Inflorescences were dipped into the agrobacterial suspension for 2-3 sec with gentle agitation. Then the plants were covered for 16-24 h with a translucent plastic lid ebfore culturing at standard conditions. Seeds were harvested and the next generation was selected with the appropriate antibiotic.

Hairy root transformation

P. hybrida W115 seeds were surface-sterilized (see above) and seedlings were grown on MS medium. Plants were transformed with *Agrobacterium rhizogenes* strain Arqua1 (Quandt et al., 1993) as described (Chabaud et al., 2006). Briefly, *A. rhizogenes* was grown for two days at 28°C on solid LB medium with the appropriate selective antibiotics. Petunia leaves were cut in pieces and dipped carefully on Petri plates containing bacterial suspension. Inoculated leaf discs were cultured on Petri plates with M medium (Bécard and Fortin, 1988) for two to four days under conditions that favor agrobacterial growth, i.e. dark conditions and temperature close to 28C.

Then leaf discs were transferred to Petri plates with selective medium (M Medium with appropriate antibiotics) for four weeks. From the selective step onwards, the plant material was grown at day/night 25/21°C on plates with 250 µg·ml^{-1} cefotaxime

or carbenicillin. Hairy roots emerged mainly from the mid vein and after three to five weeks they were cut and transferred onto fresh Petri plates with selective medium. Then hairy roots formed ROC that were self-propagated every four to six weeks on fresh selective medium.

Protein extraction and microsomal membrane fractionation

Small scale preparation: Plant material was fractionated according to (Abas and Luschnig, 2010). Briefly, tissues were ground with a mortar and pestles using liquid nitrogen and stored at -80 °C until use. Ca. 150 mg plant tissue were resuspended in 225 µl of 1.5x Buffer E and transferred to a tube containing an equal volume of an equilibrated suspension of 5 % (w/v) PVPP in buffer P. The tube was vortexed and incubated for 5 min. The pre-clearance centrifugation step was performed for 3 min. at 600g. The supernatant was collected and the pellet was resuspended in additional 225 µl buffer E and centrifuged again for 3 min. at 600g. The supernatants were pooled and this cleared homogenate was diluted with ddH2O to a final sucrose concentration of 12 %. The diluted supernatant was then divided into portions of 150-200 µl and centrifuged for 1.5 h at 21000g. The soluble fraction was collected and the membrane pellet washed with 150 µl Buffer W. The resuspended pellet was centrifuged for 45 min. at 21000g and the supernatant was discarded. During the extraction steps all buffers and samples were kept on ice and all centrifugation steps were performed at 4 °C.

Large scale preparation: Large scale microsomal membrane fractions was performed as described (Fabregas et al., 2013). Briefly, 5 g of previously ground tissue was resuspended in 10 ml cold lysis buffer and filtrated through 8 layers of cheese cloth. The pre-clearance centrifugation step was performed at 6000*g* for 20 min. and then the supernatant was collected. If the supernatant was not fully cleared, the centrifugation step was repeated. The cleared homogenate was centrifuged for 2 h at 100.000*g*. The supernatant was collected and the fractions were analyzed by Western Blot analysis or kept for solubilization tests.

Solubilization of membrane pellet fraction

For solubilization tests the membrane pellet was resuspended in according IP-Buffers containing a range of different detergents. The pellet was dissolved by vortexing and using a dounce homogenizer if not stated otherwise. The quantity of buffer used for resuspension of the membrane pellet depended on the initial tissue input and pellet yield. Small scale membrane pellets were resuspended in 30 µl and large scale membrane pellets from 5 g initial tissue were resuspended in 500 µl. To mimic following IP conditions the pellet was incubated for 1 h at 4 °C on a wheel. The membrane pellet was separated from solubilized proteins by centrifugation for 30 min. at 21000g.

Western blot analysis

SDS gel electrophoresis was performed as described (Laemmli, 1970). Proteins from the gel were transferred onto a nitrocellulose or PVDF membrane as described (Towbin et al., 1979) with a modified transfer buffer containing ethanol instead of methanol. Fusion proteins were detected with monoclonal GFP-antibody (Sigma-Aldrich) diluted 1:1000 in blocking solution. Secondary alkaline phosphatase conjugated anti-mouse antibody was diluted 1:3000 in blocking solution. Peroxidase activity was revealed according to the manufacturer.

Buffers

Lysis Buffer (50 ml)
(Fabregas *et al.*, 2013, modified)

2 M TRIS pH 7.5	500 µl	20 mM
5 M NaCl	1.5 ml	150 mM
0.5 M EDTA	100 µl	1 mM
50 % Glycerol	20 ml	20 %
20 mM PMSF	2.5 ml	1 mM
5x Protease Inhibitor	10 ml	1 x
H_2O	15.4 ml	

Buffer P (10 ml)

2 M TRIS pH 7.5	1 ml	200 mM
Sucrose	4 g	40 % (w/v)
0.5 M EDTA	400 µl	20 mM
500 mM KCl	200 µl	10 mM

Fill up with ddH_2O to the total volume of 10 ml.

Buffer E (10 ml)

	1 x		1. 5x	
2 M TRIS pH 7.5	500 µl	100 mM	750 µl	150 mM
Sucrose	2.5 g	25 % (w/v)	3.57 g	37.5 % (w/v)
50 % Glycerol	1 ml	5 %	1.5 g	7.5 %
0.5 M EDTA	200 µl	10 mM	300 µl	15 mM
100 mM DTT	100 µl	1 mM	150 µl	1.5 mM
0.5 M KCl	100 µl	5 mM	150 µl	7.5 mM
20 mM PMSF	500 µl	1 mM	750 µl	1.5 mM
5 x Protease Inhibitor	2 ml	1 x	3 ml	1.5 x

Fill up with ddH_2O to the total volume of 10 ml.

Buffer W (10 ml)

2 M TRIS pH 7.5	100 µl	20 mM
0.5 M EDTA	100 µl	5 mM
20 mM PMSF	500 µl	1 mM
5x Protease Inhibitor	2 ml	1 x
ddH_2O	7.3 ml	

Solubilization Buffer A (10 ml)

(GFP Trap®, modified)

2 M TRIS pH 7.5	500 µl	10 mM
5 M NaCl	3 ml	150 mM
0.5 M EDTA	100 µl	0.5 mM
20 mM PMSF	500 µl	1 mM
5x Protease Inhibitor	2 ml	1 x
H_2O	3.9 ml	

Solubilization Buffer B (10 ml)

(Roppolo *et al.*, 2011, modified)

1 M HEPES pH 7.9	500 µl	50 mM
5 M NaCl	300 µl	150 mM
500 mM EDTA	100 µl	5 mM
2 M Sucrose	1.5 ml	300 mM
10 mM Potassium acetate	0.1 ml	10 mM
20 mM PMSF	500 µl	1 mM
5 x Protease Inhibitor	2 ml	1 x
H_2O		

Solubilization Buffer C (10 mL)
(Fabregas *et al.,* 2013, modified)

2 M TRIS pH 7.5	50 µl	10 mM
5 M NaCl	300 µl	150 mM
0.5 M EDTA	20 µL	1 mM
50 % Glycerol	2 ml	10 %
20 mM PMSF	500 µl	1 mM
5x Protease Inhibitor	2 ml	1 x
H_2O	5.13 ml	

Abas L, Luschnig C (2010) Maximum yields of microsomal-type membranes from small amounts of plant material without requiring ultracentrifugation. Analytical Biochemistry **401:** 217-227

Bécard G, Fortin JA (1988) Early events of vesicular arbuscular mycorrhiza formation on Ri T-DNA transformed roots. New Phytologist **108:** 211-218

Bechtold N, Ellis J, Pelletier G (1993) *In planta Agrobacterium*-mediated gene transfer by infiltration of adult *Arabidopsis thaliana* plants. Comptes Rendus De L Academie Des Sciences Serie Iii-Sciences De La Vie-Life Sciences **316:** 1194-1199

Chabaud M, Boisson-Dernier A, Zhang J, Taylor CG, Yu O, Barker DG (2006) *Agrobacterium rhizogenes*-mediated root transformation. *In* Medicago truncatula Handbook,

Fabregas N, Li N, Boeren S, Nash TE, Goshe MB, Clouse SD, de Vries S, Cano-Delgado AI (2013) The BRASSINOSTEROID INSENSITIVE1-LIKE3 signalosome complex regulates *Arabidopsis* root development. Plant Cell **25:** 3377-3388

Laemmli UK (1970) Cleavage of structural proteins during assembly of head of bacteriophage-T4. Nature **227:** 680-&

Quandt HJ, Puhler A, Broer I (1993) Transgenic root nodules of *Vicia hirsuta* - A fast and efficient system for the study of gene expression in indeterminate-type nodules. Molecular Plant-Microbe Interactions **6:** 699-706

Towbin H, Staehelin T, Gordon J (1979) Electrophoretic transfer of proteins from polyacrylamide gels to nitrocellulose sheets - procedure and some applications. Proceedings of the National Academy of Sciences of the United States of America **76:** 4350-4354

Zhang XR, Henriques R, Lin SS, Niu QW, Chua NH (2006) *Agrobacterium*-mediated transformation of *Arabidopsis thaliana* using the floral dip method. Nature Protocols **1:** 641-646
